# ANTHOCYANIDIN REDUCTASE promotes physical dormancy in *Medicago truncatula* seeds

**DOI:** 10.1093/plphys/kiaf525

**Published:** 2025-10-17

**Authors:** Zhaozhu Wen, Juan Du, Maofeng Chai, Xuran Lu, Huancheng Liu, Qinyan Bao, Yanbo Zhang, Hanyu Huo, Zicheng Wang, Jiangqi Wen, Kirankumar S Mysore, Juan Sun, Zhifei Zhang, Zeng-Yu Wang

**Affiliations:** College of Agronomy, Hunan Agricultural University, Changsha 410128, China; College of Grassland Science, Qingdao Agricultural University, Qingdao 266109, China; Institute for Agricultural Biosciences, Oklahoma State University, Ardmore, OK 73401, USA; College of Grassland Science, Qingdao Agricultural University, Qingdao 266109, China; College of Grassland Science, Qingdao Agricultural University, Qingdao 266109, China; College of Grassland Science, Qingdao Agricultural University, Qingdao 266109, China; College of Grassland Science, Qingdao Agricultural University, Qingdao 266109, China; College of Grassland Science, Qingdao Agricultural University, Qingdao 266109, China; College of Grassland Science, Qingdao Agricultural University, Qingdao 266109, China; College of Grassland Science, Qingdao Agricultural University, Qingdao 266109, China; Institute for Agricultural Biosciences, Oklahoma State University, Ardmore, OK 73401, USA; Institute for Agricultural Biosciences, Oklahoma State University, Ardmore, OK 73401, USA; College of Grassland Science, Qingdao Agricultural University, Qingdao 266109, China; College of Agronomy, Hunan Agricultural University, Changsha 410128, China; College of Grassland Science, Qingdao Agricultural University, Qingdao 266109, China

## Abstract

Physical dormancy, or hardseededness, refers to a type of dormancy in which seeds cannot germinate due to seed coat impermeability. Physical dormancy broadly exists in seed plants, especially in leguminous species, and plays an essential role in maintaining the durability of natural seed banks. However, physical dormancy restricts the utilization of leguminous seeds in agricultural production. Seeds of the leguminous model plant *Medicago truncatula* show typical physical dormancy. In this study, we report a function of anthocyanidin reductase (ANR) in controlling *M. truncatula* seed physical dormancy. The *ANR* gene was mostly highly expressed in the *M. truncatula* seed coat. Loss-of-function mutations in *ANR* resulted in the absence of hardseededness, allowing the seed to absorb water quickly without scarification. The content of glycolipids, especially MDGD, was significantly decreased in the seed coat of the *anr* mutant. A large increase in the levels of the most abundant flavonoids (flavonoid-3-*O*-glucosides) was also observed in the *anr* mutant seed coat. Knockout of the upstream genes *transparent testa 8* (*TT8*), *flavonoid 3′,5′-hydroxylase 1* (*F3′5'H1*), or *anthocyanidin synthase* (*ANS*) hindered flavonoid-3-*O*-glucoside accumulation. Accordingly, mutating these genes in the *anr* background (*anr tt8*, *anr f3′5′h1,* and *anr ans*) restored seed physical dormancy. These results indicate that proanthocyanidins are not directly associated with hardseededness. Rather, an excessive accumulation of flavonoid-3-*O*-glycosides and a reduction in lipids are associated with seed physical dormancy. This study provides information regarding the molecular and biochemical mechanisms underlying physical dormancy.

## Teaser

The function of ANTHOCYANIDIN REDUCTASE in controlling seed physical dormancy is revealed in the model legume *Medicago truncatula*, which has typical hardseededness.

## Introduction

Seed dormancy is an adaptive trait that enables long-term survival of embryos of angiosperm plants under adverse environmental conditions (Koornneef et al. 2002; Bentsink and Koornneef 2008). Over the course of evolution, plant seeds have evolved various forms of dormancy to adapt to various climates and environments to maintain their viability (Finch-Savage and Leubner-Metzger 2006). Seed dormancy has been assigned to 5 classes: physiological dormancy, physical dormancy, morphological dormancy, morphophysiological dormancy, and physical plus physiological dormancy (Baskin and Baskin 2004, 2014).

Physical dormancy, also known as hardseededness, is a dormancy phenomenon in which seed germination is prevented due to the impermeability of the seed coat (Baskin et al. 2000; Koizumi et al. 2008; Weitbrecht et al. 2011; Radchuk and Borisjuk 2014). Physical dormancy is present in at least 18 families of angiosperms and is possibly caused by the presence of phenolics and suberin-impregnated layers of palisade cells (Baskin and Baskin 2014). Gymnosperm seeds do not show physical dormancy because their seed coat does not have a water-impermeable palisade layer (Smýkal et al. 2014). The presence of the seed coat prolongs seed vigor, especially in seeds harvested under field conditions (Hartwig and Potts 1987). The seed coat blocks various unfavorable substances in the surrounding environment, improves the resistance of seeds to pathogens, and prevents microbial attack (Yaklich and Kulik 1987; Kulik and Yaklich 1991; Roy et al. 1994).

On the other hand, seed physical dormancy may negatively impact agricultural production. The physical dormancy of seeds usually results in poor quality and increases costs for processing vegetable oil and other products (Yoshikawa et al. 2014). There is also a weed risk associated with hardseededness in the use of wild or less domesticated species as cover crops because their seeds lie dormant in the soil for years. The loss of seed physical dormancy is crucial to the domestication of cultivated leguminous crops, and it is considered one of the key indices in the domestication process (Ladizinsky 1979; Sedivy et al. 2017). Therefore, investigation of seed physical dormancy is important to plant domestication, plant breeding (especially legumes), and seed production.

Compared with physiological dormancy, which has been well studied in *Arabidopsis thaliana* and grains, studies on physical dormancy have mainly focused on the morphology, structure, and chemical composition of the seed coat, including phenolic content and stratum corneum composition, with only a few articles published on its molecular aspects (Baskin and Baskin 2004; Finkelstein et al. 2008; Graeber et al. 2012; Radchuk and Borisjuk 2014; Hradilová et al. 2017). In the molecular analysis of soybean (*Glycine max*) hardseededness, it has been shown that *hard-seededness 1* (*GmHs1-1*) is primarily expressed in the Malpighian layer of the seed coat and affects hardseededness, it encodes a calcineurin-like metallophosphoesterase transmembrane protein in soybean (Sun et al. 2015). Another gene, *GmqHS1*, which is adjacent to *GmHs1-1* on soybean chromosome 2, was also found to regulate physical dormancy (Jang et al. 2015). The *GmqHS1* gene was transferred to the water-permeable soybean variety “Kariyataka,” resulting in the accumulation of β-1,4-glucan on the outer side of the palisade layer cells and producing hard seeds (Jang et al. 2015).

The seed of the model legume *Medicago truncatula* exhibits a typical hardseededness phenotype, thus making this species an ideal model for studying physical dormancy (Bolingue et al. 2010; Chai et al. 2016, 2021). A class II KNOTTED-like homeobox (KNOX4) has been shown to make the palisade cuticle layer dysfunctional in *M. truncatula* (Chai et al. 2016). A β-ketoacyl-CoA synthase (KCS12) is an enzyme that controls the production of very long-chain lipid (VLCFA) species in the seed coat, the gene coding for KCS12 is directly regulated by the KNOX4 transcription factor (Chai et al. 2021).

Flavonoids play important roles in plant survival, color, and reproduction. Flavonoids are one of the largest groups of plant specialized metabolites, and the main classes of these phenylpropanoid pathway derivatives include flavonols, anthocyanins, and proanthocyanidins (PAs) (Xu et al. 2015). Anthocyanidin reductase (ANR), an enzyme of the phenylpropanoid pathway that participates in the biosynthesis of PAs, converts anthocyanidins to their corresponding 2,3*-cis*-flavan-3-ols; overexpression of the *ANR* gene results in the loss of anthocyanidins and accumulation of PAs (Xie et al. 2003; Jun et al. 2021). PAs, also known as condensed tannins (CTs), are oligomers and polymer end products of the flavonoid biosynthetic pathway and are present in seeds, fruits, leaves, and epidermal layers of a large number of plant species (Balentine et al. 1997; Prior and Gu 2005; Donaldson et al. 2006). PAs are also the main quality factor of forage crops. They are combined with forage protein to prevent the ruminant bloat problem and improve nitrogen nutrition by increasing the amount of rumen-passing protein (Douglas et al. 2010). Anthocyanidins are not only converted into PAs under the action of ANR but also form anthocyanins through glycosylation, methylation, and acylation reactions under the action of various UDP-glucosyltransferases. This process makes anthocyanins more stable in property, more complex in structure, and more abundant in color. For plants, anthocyanins play an important role in stress response. Abiotic stress stimuli, such as UV, low temperature, and drought stress, lead to the accumulation of anthocyanins in plants (Gould et al. 2010). For animal and human health, anthocyanins play an important role in protecting human vision, improving blood sugar balance, and reducing the occurrence of hyperlipidemia due to their strong antioxidant activity (Kalt et al. 2008). A wealth of valuable information has been accumulated based on studies of ANR enzyme kinetics, expression regulation, and gene function in various plant species, such as *Arabidopsis*, *Vitis vinifera,* and *M. truncatula* (Xie et al. 2004; Gargouri et al. 2010; Dixon and Sarnala 2020). However, to date, no information is available on the function of ANR in physical dormancy.

In this study, we discovered a function of ANR in physical dormancy. Loss-of-function of ANR in *M. truncatula* resulted in an absence of hardseededness, allowing the seed to absorb water quickly without scarification. Phenotypical, microscopic, biochemical, genetic, and molecular analyses showed that the permeability of the seed coat of the *anr* mutant is associated with reduced lipid content and excessive accumulation of flavonoid-3-*O*-glucosides. These results revealed that ANR plays an important role in seed coat development, and we proposed a simplified functional model of *ANR* in regulating seed physical dormancy.

## Results

### Expression analysis of *ANR* in *M. truncatula*

**Figure 1.**
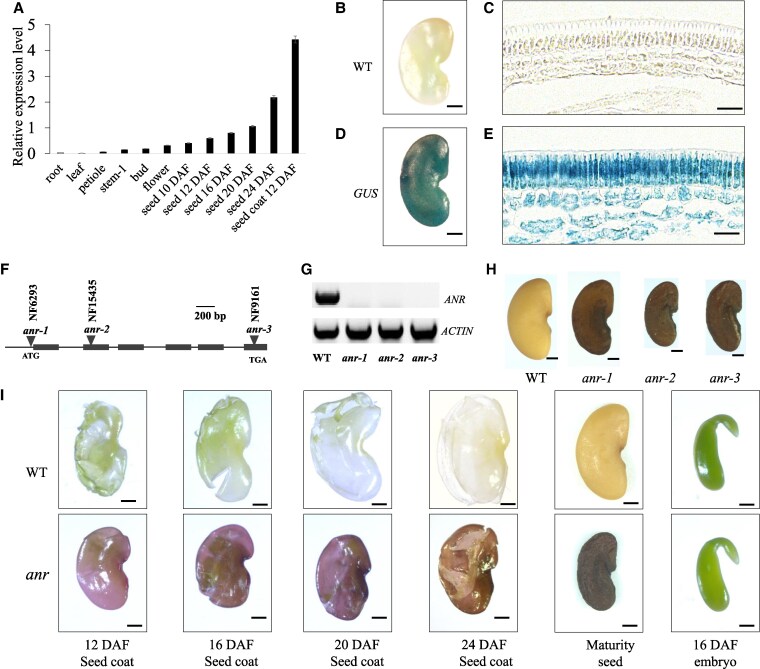
Isolation and characterization of *anr* mutant seeds in *Medicago truncatula*. **A)** RT-qPCR analysis of *ANR* expression in different plant organs in 3 independent experiments, error bars represent standard deviation (SD). **B–E)** GUS staining of seed coat at 16 DAF of WT and different stages of transgenic *M. truncatula* plants carrying the *proANR::GUS* construct, scale bar = 500 *μ*m. **C, E)** Cross-section of GUS stained seed coat, scale bar = 20 *μ*m. **F)** Schematic representation of *ANR* gene structure and *Tnt1* insertion sites, filled black boxes represent exons and lines between them denote introns. **G)** RT-PCR analysis of *MtANR* transcript levels in WT and *anr* mutant, *ACTIN* used as the control. **H)** Morphologies of mature seeds from WT and *anr* mutants, scale bar = 500 *μ*m. **I)** Time-course analysis of seed coat development in WT and *anr* mutant, scale bar = 500 *μ*m.

Based on the *M. truncatula* gene expression atlas (INRAE/CNRS Medicago Expression Atlas), the expression of *ANR* (ecotype A17, Mtr.44985.1. S1_at) is higher in the seed coat than in other tissues and organs (Supplementary Fig. S1). To confirm and to further characterize the expression pattern of the *ANR* gene, total RNA was isolated from the roots, leaf, petiole, stem, bud, flower, seed (12, 16, 20, and 24 d after flowering, DAF), and seed coat (12 DAF). Reverse transcription quantitative polymerase chain reaction (RT-qPCR) analysis showed that *ANR* had the highest expression in the seed coat (Fig. 1A).

To determine whether there is an association between the *ANR* expression pattern and seed coat development, we introduced the *β-glucuronidase* reporter gene driven by the *ANR* promoter (*proANR::GUS*) into wild-type *M. truncatula* and analyzed the transgenic plants at 12, 16, 20, and 24 DAF. Strong GUS activity was detected in seed coats (Fig. 1, B to E). These results suggest that *ANR* plays an essential role in seed coat development.

ANR orthologs in various species were identified using BLASTN in NCBI and Phytozome and used for bioinformatics analysis. Analysis of deduced amino acid sequences revealed that the MtANR protein contained 335 amino acids and shared a high identity with the amino acid sequence of *Medicago sativa* ANR (MsANR, 96.13%) (Supplementary Fig. S2). The MtANR protein was also highly similar to its putative orthologs in *Trifolium pratense* (91.02%), *Vicia faba* (89.25%), *Trifolium repens* (89.82%), and *Cicer arietinum* (86.27%), indicating that ANR is highly conserved in legume species (Supplementary Fig. S2). Phylogenetic analysis showed that MtANR was clustered closely to MsANR (Supplementary Fig. S3).

### Identification and characterization of *ANR* mutants in *M. truncatula*

To analyze the role of *ANR* in seed coat, 3 mutant alleles, *anr-1*, *anr-2* and *anr-3*, were identified from the *Tnt1* retrotransposon-tagged mutant population of *M. truncatula* (Fig. 1F). Analysis of the mutants revealed *Tnt1* insertion sites of *anr-1* (*Tnt1* inserted before base 58), *anr-2* (*Tnt1* inserted after base 774) and *anr-3* (*Tnt1* inserted after base 2480) (Fig. 1F). To examine the expression levels of *ANR* in the *Tnt1* insertion lines, total RNA was isolated from the wild type and *anr-1*, *anr-2,* and *anr-3*. RT-PCR analysis demonstrated that transcription of *ANR* was abolished in the mutants (Fig. 1G).

The mature seeds of the 3 *anr* mutants were reddish-brown while the wild-type seeds were yellow (Fig. 1H). The seed coat and embryo of immature seeds were separated at different developmental stages, and pigmentation was observed in the seed coat but not in the embryo (Fig. 1I). Based on our observation, the color change of the seed coat of the *anr* mutants became noticeable at 2 DAF. As seeds continued to develop, the seed coat color of the *anr* mutants became progressively darker (Fig. 1I).

### The role of ANR in controlling seed physical dormancy

**Figure 2.**
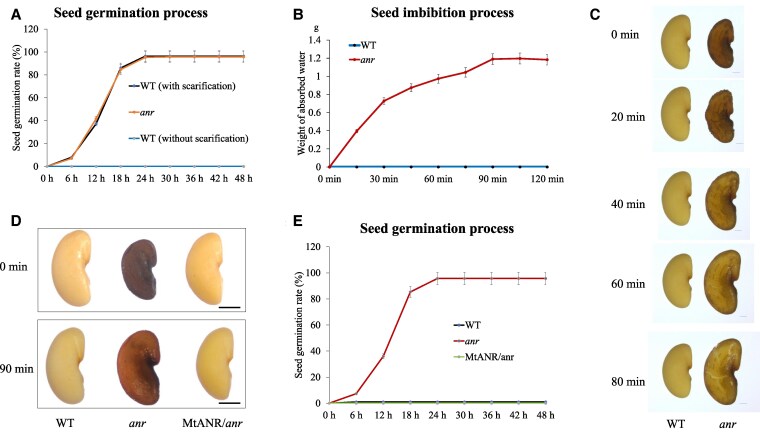
Characterization of *anr* mutant seeds lacking physical dormancy in *M. truncatula*. **A)** Germination process of WT and *anr* mutant (*anr*-2) seeds, with and without scarification treatment, in sterile water for 2 d. **B)** Weight of absorbed water of WT and *anr* mutant at multiple timepoints. min, minutes. **C)** Representative photographs of WT and *anr* mutant seeds during the imbibition process in sterile water, scale bar = 500 *μ*m. The WT seed from Fig. 2C is the same as that shown in Fig. 1H. **D)** Mature seed morphologies of WT, *anr* mutant, and the complementation line in the *anr* mutant background, scale bar = 500 *μ*m. **E)** Germination process of WT, *anr* mutant, and the complementation line seeds without scarification treatment. Error bars represent SD in this figure.

We investigated the seed imbibition process and found that the mutant seeds imbibed water in 10 min without any scarification treatment (Supplementary Movies S1 and S2). After placing seeds in water, more than 90% of the mutant seeds germinated without scarification in 24 h, whereas the wild-type (WT) seeds did not show any sign of germination (Fig. 2A). After scarification treatment with sandpaper, the WT seeds showed a similar germination rate as the mutant seeds (Fig. 2A). Seed swelling was observed in the mutants after only 20 min in water, whereas the WT seeds remained static after days in water (Fig. 2, B and C, Supplementary Fig. S4A). These results demonstrated that the mutant seeds lost physical dormancy.

To further confirm that the seed phenotype was caused by the loss-of-function of ANR, we performed a complementation experiment. The *ANR* coding sequence driven by its native promoter was transformed into a homozygous *anr-3* line. RT-PCR analysis showed that transcription of *ANR* was detected in the complementation lines (Supplementary Fig. S4B). Seeds harvested from the transgenic plants exhibited a similar phenotype to that of the WT seeds in water (Fig. 2D). Seed germination rate and permeability analysis were also performed using the *anr* mutant, the complementation lines, and the WT., the transferred *ANR* gene completely restored physical dormancy in seeds of the complementation lines (Fig. 2E and Supplementary Fig. S4C).

### Structural difference of the seed coat between the *anr* mutants and the wild type

**Figure 3.**
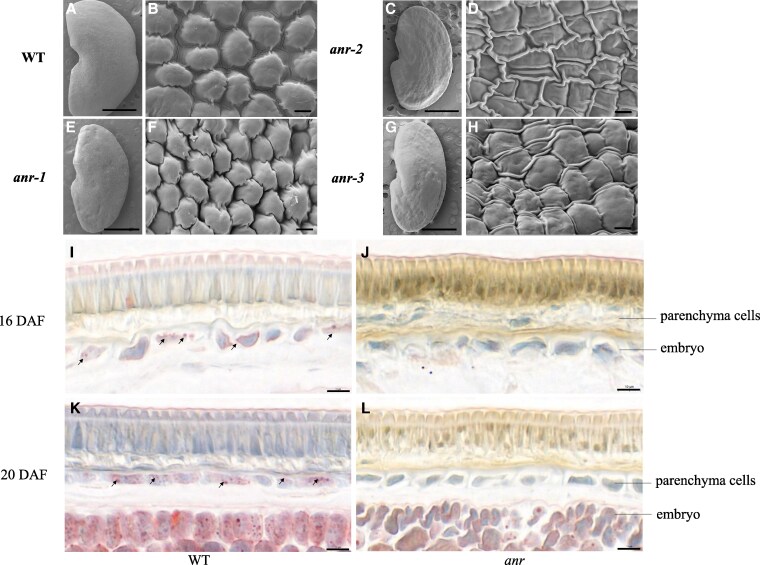
Structural difference of the seed coat between the *anr* mutants and the WT in *M. truncatula*. **A–H)** SEM observation of mature seed coats from WT and *anr* mutants in *M. truncatula*. **A, C, E,** and **G)** Scale bar = 1 mm, **B**, **D**, **F,** and **H**: Scale bar = 2 *μ*m. **I–L)** Cross-sections of WT **(I**, **K)** and *anr* mutant **(J**, **K)** stained with Oil red O at 16 and 20 DAF, scale bar = 10 *μ*m. The arrow points to visible lipid droplets in **I** and **K**.

Seed coat surface morphology of the *anr* mutant and the WT was examined by scanning electron microscopy (SEM). In the WT, the surface was very smooth with regularly arranged dome-like epidermal cells (Fig. 3, A and B). In contrast, the mutant seed surface was wrinkled with an irregular shape, and the intercellular space was shrunken and more compact (Fig. 3, B to H).

By careful observation of the *anr*-1 mutant, it was found that the seeds were darker near the hilum. To further analyze the structural differences of the seed coat, 3 parts of the seed were observed using SEM: (i) the edge away from the seed hilum, (ii) the middle of the seed, and (iii) near the hilum. In the WT, there was little difference in seed coat surface structure at these 3 positions (Supplementary Fig. S5). However, in the mutant seeds, the closer to the hilum, the smaller the gap between epidermal cells (Supplementary Fig. S5). In mutants *anr*-2 and *anr*-3, the epidermal cells near the hilum lost their original shape and were pressed into a line shape (Supplementary Fig. S5).

To compare cellular structure between the WT and the *anr* mutants, longitudinal sections of the seed coat at 20 DAF and maturity were stained with 0.05% Toluidine blue O. The seed coat longitudinal structure of the *anr* mutants were relatively intact, and there were no obvious differences in the cuticle layer, bright line, palisade layer cells, hourglass cells, and parenchyma cells compared with the WT (Supplementary Fig. S6).

Cryosections of the seed coat at 16 and 20 DAF were stained with oil red O, a fat-soluble dye that can specifically turn lipids (such as triglycerides) into red color in tissues. In the WT, multiple visible lipid droplets were easily observed in the parenchyma cells, while almost no lipid droplets were observed in the *anr* mutants (Fig. 3, I to L).

### Changes in lipid accumulation in the seed coat of the *anr* mutant

**Figure 4.**
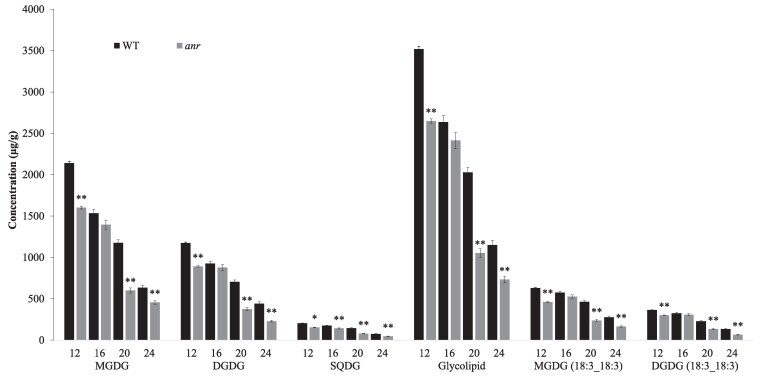
Content of lipid monomers in WT and *anr* mutant of *M. truncatula*. MGDG: monogalactosyldiacylglycerol; DGDG: digalactosyldiacylglycerol; SQDG: sulfoquinovosyldiacylglycerol. The vertical axis represents concentration (*μ*g/g). Error bars represent SD, asterisks indicate significant differences between WT and *anr* mutant using 2-tailed unpaired Student's *t*-test (*P < 0.05; **P < 0.01).

To assess the impact of *ANR* mutation on lipid biosynthesis, the seed coat of the *anr* mutant and the WT were analyzed using a UPLC-ESI-MS/MS system at different developmental stages (12, 16, 20, and 24 DAF). Compared with the WT, contents of monogalactosyldiacylglycerol (MGDG), digalactosyldiacylglycerol (DGDG), and sulfoquinovosyldiacylglycerol (SQDG) showed a decreasing trend in the *anr* mutant at different stages, particularly at 12 and 20 DAF, while the remaining 28 subclasses of lipids did not show a consistent change in the mutant (Supplementary Table S1). The content of glycolipids (MGDG, DGDG, and SQDG) was decreased by 24.72%, 8.42%, 48.08%, and 36.32% in the *anr* mutant seed coat at 12, 16, 20, and 24 DAF, respectively (Fig. 4). The chain lengths of MGDG, DGDG, and SQDG were mainly 32 to 36 °C, belonging to VLCFA (Supplementary Fig. S7).

At the individual monomer level, the content of MGDG (18:3_18:3) and DGDG (18:3_18:3) was significantly decreased in the *anr* mutant at 12, 20, and 24 DAF (Fig. 4, Supplementary Table S2). Compared with the WT, the content of MGDG (18:3_18:3) decreased 26.70%, 8.34%, 49.17%, and 40.59%, respectively, at 12, 16, 20, and 24 DAF; the content of DGDG (18:3_18:3) decreased by 17.34%, 5.27%, 42.06%, and 51.22%, respectively, at 12, 16, 20, and 24 DAF (Fig. 4).

### Changes in flavonoid accumulation in the *anr* mutant seed coat

Flavonoid accumulation in the seed coat was analyzed using the UPLC-ESI-MS/MS system at different developmental stages. Three categories of flavonoids were found in the seed coat at 12, 16, 20, and 24 DAF: anthocyanins (cyanidins, petunidins, malvidins, pelargonidins, peonidins, and delphinidins), PAs, and other flavonoids. Compared with the WT, the contents of anthocyanins and other flavonoids significantly increased in the *anr* mutant, while the contents of PAs significantly decreased (Supplementary Fig. S8).

**Figure 5.**
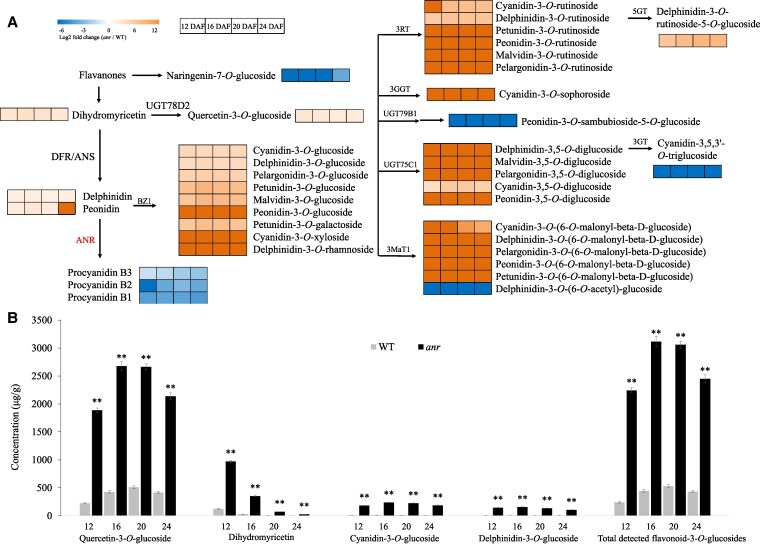
Different flavonoids accumulated during seed coat development in *M. truncatula*. **A)** Heatmap of differential metabolites in the flavonoid synthesis pathway compared with WT; Orange represents increase, dark orange represents new compounds in *anr* mutant, blue represents decrease; **B)**Content of the 4 highest flavonoids. Error bars represent SD, asterisks indicate significant differences between WT and *anr* mutant using 2-tailed unpaired Student's *t*-test (*P < 0.05; **P < 0.01). DFR, dihydroflavonol 4-reductase; UGT78D2, flavonol 3-*O*-glucosyltransferase; BZ1, anthocyanidin 3-*O*-glucosyltransferase; 6''-*O*-acyltransferase; 3RT, anthocyanidin 3-*O*-rutinosyltransferase; 3GGT, anthocyanidin 3-*O*-glucoside 2''-*O*-glucosyltransferase; UGT75C1, anthocyanidin 3-*O*-glucoside 5-*O*-glucosyltransferase; UGT79B1, anthocyanidin 3-*O*-glucoside 2'''-*O*-xylosyltransferase; 3MaT1, anthocyanin 3-*O*-glucoside-6''-*O*-malonyltransferase; 5GT: cyanidin 3-*O*-rutinoside 5-*O*-glucosyltransferase; 3′GT: anthocyanin 3′-*O*-beta-glucosyltransferase.

Thirty-eight of the identified flavonoid metabolites showed a significant difference between the *anr* mutant and the WT at 4 stages (Supplementary Fig. S9). Sixteen anthocyanins were only present in the *anr* mutant but undetectable in the WT (dark orange in the heatmap), which may be related to the *anr* mutant seeds turning reddish-brown (Fig. 5A). The content of 31 flavonoids was higher in the *anr* mutant seed coat than in the WT, including some uncommon and low levels of anthocyanins such as cyanidin-3-*O*-xyloside and delphinidin-3-*O*-rhamnoside (Fig. 5A).

The 4 most abundant differential flavonoids in the *anr* mutant were quercetin-3-*O*-glucoside, dihydromyricetin, cyanidin-3-*O*-glucoside and delphinidin-3-*O*-glucoside, accounted for up to 48%, 17%, 4%, and 3% of total flavonoid content (Fig. 5B). Compared with the WT, the content of quercetin-3-*O*-glucoside, dihydromyricetin, cyanidin-3-*O*-glucoside, and delphinidin-3-*O*-glucoside in the mutant increased 4.13–7.49, 6.20–10.98, 19.52–22.19, and 17.00–22.33 fold, respectively, at different stages (Fig. 5B). The content of total detected flavonoid-3-*O*-glucosides in the seed coat of the *anr* mutant accounted for up to 55% of total detected flavonoid content and increased 4.68–8.4 fold compared with the WT (Fig. 5B).

In contrast, proanthocyanidin B1, proanthocyanidin B2, and proanthocyanidin B3 were lower in the *anr* mutant seed coat than in the WT. Proanthocyanidin B2, one of the *ANR* synthetic products, showed the most drastic reduction in the *anr* mutant, which was more than 61-fold lower relative to the WT seed coat (Fig. 5A). Therefore, mutation of *ANR* promoted the accumulation of flavonoid compounds, including some uncommon anthocyanins, and blocked the conversion of anthocyanidins to PAs.

### Genetic analysis of seed physical dormancy in double mutants

**Figure 6.**
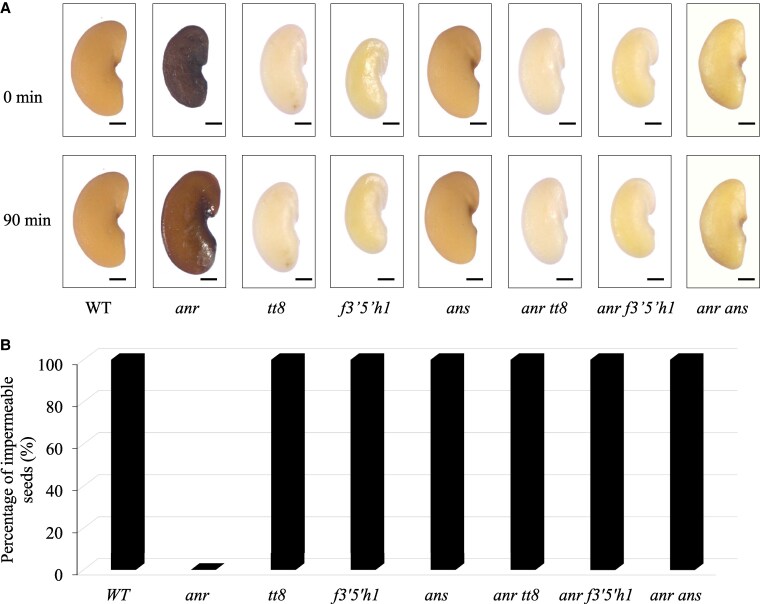
Analysis of seed physical dormancy in double mutants in *M. truncatula*. **A)** Seed permeability analysis of WT, *anr*, *tt8*, *f3′5′h1*, *ans*, *anr tt8*, *anr f3′5′h1,* and *anr ans* mutant seeds, scale bar = 500 *μ*m. **B)** Seed permeability analysis of WT and *anr*, *tt8*, *f3′5′h1*, *ans*, *anr tt8*, *anr f3′5′h1,* and *anr ans* mutants.

To further investigate the role of *ANR* in seed coat formation, the *anr* mutant was crossed with 3 different *M. truncatula* mutants: *tt8*, *f3′5′h1,* and *ans*. The mutants *tt8* and *f3′5′h1* did not synthesize anthocyanins and PAs, the seed coat was transparent and impermeable (Fig. 6). The *ans* mutant did not produce anthocyanidins but PAs in the seed coat, and the seed coat was impermeable, and its color was normal. The F_1_ generation seeds harvested after crossing *anr* with each of the *tt8*, *f3′5′h1,* and *ans* mutants showed the same genetic characteristics as the maternal genotype because the seed coat was developed from the integument (Supplementary Fig. S10). The seed coat color of the F_2_ generation was the same as that of the wild type (Supplementary Fig. S10). During seed coat permeability analysis of the F_3_ generation seeds, the *anr tt8*, *anr f3′5′h1,* and *anr ans* double mutant seeds did not imbibe water even after days in sterile water, while the *anr* mutant seeds imbibed water (Fig. 6B). Namely, the *anr tt8* and *anr f3′5′h1* double mutant seeds were transparent and showed physical dormancy, the *anr ans* double mutant seeds showed normal color and physical dormancy.

### Lipid and flavonoid analysis in the seed coat of the *anr tt8* double mutant

**Figure 7.**
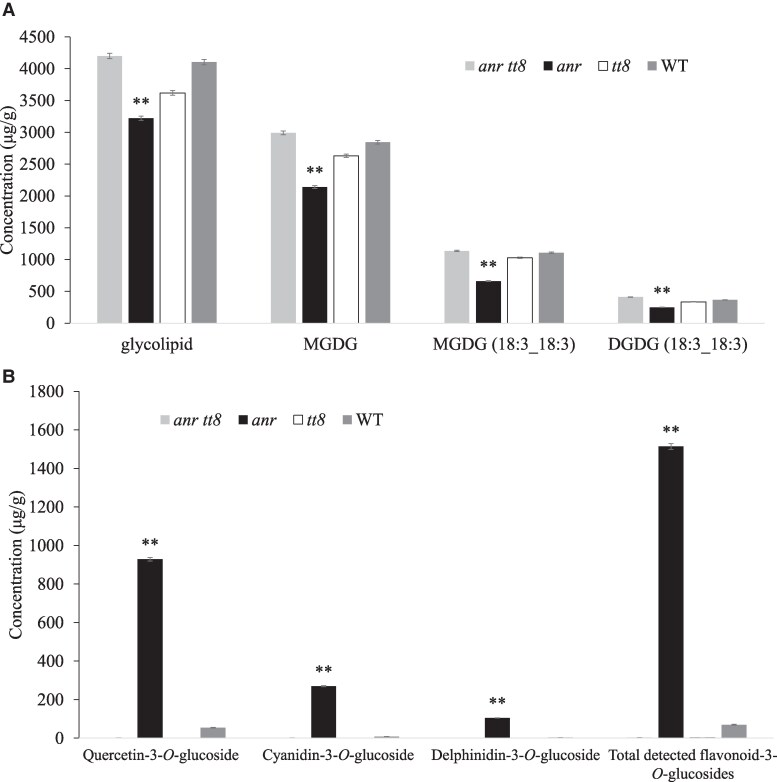
The content of lipid (A) and flavonoid (B) in WT and mutants of *M. truncatula*. The vertical axis represents concentration (*μ*g/g). Error bars represent SD, asterisks indicate significant differences using a 2-tailed unpaired Student's *t*-test in this figure (*P < 0.05; **P < 0.01).

Seed coat of the *anr tt8* double mutant, *anr* mutant, *tt8* mutant, and WT was analyzed at 24 DAF. Significant difference in glycolipids was found between *anr* (permeable seed coat) and *anr tt8*, *tt8* as well as WT (impermeable seed coat) (Fig. 7A). The content of glycolipids in *anr* decreased 23.28%, 10.94%, and 21.46% compared with *anr tt8*, *tt8* and WT, respectively (Fig. 7A). The content of MGDG in *anr* was decreased 28.44%, 18.65%, and 24.74% compared with *anr tt8*, *tt8,* and WT, respectively (Fig. 7A). At the individual monomer level, compared with *anr tt8*, *tt8* and WT, the content of MGDG (18:3_18:3) decreased 41.90%, 35.70%, and 40.31% respectively in the *anr*, the content of DGDG (18:3_18:3) decreased 39.28%, 25.84%, and 32.02% respectively in the *anr* (Fig. 7A).

In the anr mutant, the content of quercetin-3-*O*-glucoside increased 677.78, 1701.15, and 16.23 fold, respectively, at 24 DAF compared with the *anr tt8*, *tt8,* and WT; the content of cyanidin-3-*O*-glucoside increased 183.77, 252.09, and 31.34 fold, respectively; the content of delphinidin-3-*O*-glucoside increased 1824.50, 3000.14, and 30.96 fold, respectively (Fig. 7B). The content of total detected flavonoid-3-*O*-glucosides in the anr mutant increased 478.57, 838.84, and 20.03 fold respectively at 24 DAF compared with the *anr tt8*, *tt8,* and WT (Fig. 7B). In addition, total detected PAs, including PA B1, PA B2, PA B3, PA B4, and PA C1, were drastically reduced in the *anr*, *tt8,* and *anr tt8* than in the WT (Supplementary Fig. S11).

### ANR affects the expression of lipid metabolism genes

Our preliminary experiments showed that *M. truncatula* seeds started to exhibit physical dormancy at 20 DAF. To compare the expression profiles between the *anr* mutants and the WT to gain further insights into the transcriptional regulatory network by which *ANR* affects physical dormancy, developing seed coats were collected at 12, 16, 20, and 24 DAF, RNAs were extracted from the samples, and RNA-Seq analysis was performed. More than 40 million raw reads were generated from each library, and 86% to 93% of trimmed reads were uniquely mapped to the *M. truncatula* (R108) reference genome (https://medicago.toulouse.inrae.fr/MtrunR108_HiC/) (Supplementary Table S3).

The comparison of transcript abundance in these developing seed coats uncovered 377 differentially expressed genes between the *anr* mutant and the WT, which were shared by the 4 stages (Supplementary Fig. S12A). According to gene ontology (GO) analysis, these differentially expressed genes were mainly involved in lipid and cell wall pathways, including lipid catabolic process (GO:0016042), lipid transport (GO:0006869), and cell wall biogenesis (GO:0042546) (Supplementary Fig. S13). From these pathways, 19 candidate genes related to lipids and cell walls were obtained (Supplementary Table S4). Compared with the WT, the heatmap clearly showed that the 19 candidate genes were differentially expressed in the *anr* mutant at the 4 stages (Supplementary Fig. S12B). Two lipid-related genes, LTP-2 and CYP72A67, encoding enzymes involved in lipid transfer and seed storage, were the most downregulated (more than 16-fold) in the *anr* mutant compared with the WT (Supplementary Fig. S12B and Table S4).

### ANR is involved in seed longevity

To investigate the effect of *ANR* mutation on seed longevity, seed germination tests were carried out using 3-year-old seeds and freshly harvested dry seeds. For the 3-year-old seeds, the germination rate of the *anr* mutant was significantly lower than that of the WT as well as the complementation line (Supplementary Fig. S14). For freshly harvested dry seeds, the *anr* mutant showed a similar germination rate as the WT and the complementation line (Supplementary Fig. S14).

## Discussion

As a model legume plant, *M. truncatula* has been effectively used for studying nitrogen fixation, compound leaf development, autotoxicity, and other important traits (Gou et al. 2017; Senovilla et al. 2018; Zhou et al. 2019; Du et al. 2021; Wang et al. 2022). The seeds of *M. truncatula* belong to a classic hardseededness type, making the species a suitable model for studying physical dormancy. To date, our understanding of the molecular mechanisms regulating physical dormancy is very limited. In this paper, we elucidated an essential function of ANR in physical dormancy and seed coat development.

### ANR plays a critical role in seed physical dormancy

ANR, a key enzyme affecting the synthesis of PAs and anthocyanins, has been extensively investigated in different plants (Albert et al. 1997; Xie et al. 2003; Kosonen et al. 2015; Gao et al. 2019). It has been shown that ANR has dual activity and is involved in the production and formation of (−)-epicatechin and 2,3-*cis*-leucocyanidin, which account for the presence/absence of PA polymerization and the composition of PAs across plant species (Jun et al. 2021). ANR also leads to the accumulation of 4β-(*S*-cysteinyl)-epicatechin in maize seeds with active flavonoid biosynthesis (Lu et al. 2023). By investigating sequences of about 700 accessions of adzuki bean (*Vigna angularis*), a recent study showed that *VaANR1* is associated with seed coat color evolution and possibly seed germination (Chien et al. 2025). Genome-wide association study (GWAS) in *Vigna angularis* suggested that *VaANR1* may exert a moderate impact on seed permeability and germination (Chien et al. 2025), however, the absence of relevant mutant materials precluded the experimental validation of this hypothesis.

Although *anr* mutants in *Arabidopsis* also showed a darker seed coat color when compared with the control, due to the lack of typical physical dormancy in *Arabidopsis* seeds (Baskin and Baskin 2014), the possible role of AtANR in hardedseedness has never been reported. While screening a large number of seeds in the model legume *M. truncatula,* which show typical physical dormancy, we found that *anr* mutant seeds were able to germinate without scarification. We observed that the coloration of *anr* mutant seed coat became progressively darker as the seed develops, and this color change was caused by the accumulation of anthocyanins. Further analyses confirmed the function of ANR in regulating seed coat development and physical dormancy.

### PA accumulation is not directly associated with physical dormancy

The biosynthetic pathways of PAs and anthocyanins are regulated by various factors. F3′5'H alters the type of flavonoid, converting kaempferol/dihydroquercetin to isorhamnetin/dihydrokaempferol and dihydroquercetin/myricetin, thereby affecting plant color (Zabala and Vodkin 2007; Moreau et al. 2012). The bHLH transcription factor TT8 is an important central component of the MBW complex, which regulates the biosynthesis of anthocyanins and PAs. Studies have shown that MtTT8 forms a ternary complex with MYB and MtWD40-1 to activate the *ANR* gene, and BnTT8 affects the structure of the seed coat of rapeseed (Li et al. 2016; Zhai et al. 2020). Mutants of *f3′5'h1* and *tt8* do not accumulate PAs in seed coats (Moreau et al. 2012; Li et al. 2016; Zhai et al. 2020). Anthocyanidin is formed from leucocyanidin by an iron-dependent 2-oxoglutarate-dependent dioxygenase termed anthocyanidin synthase (Saito et al. 1999). The homolog of ANS, leucoanthocyanidin dioxygenase (LDOX), is involved in parallel pathways to generate PAs in *M. truncatula* (Liu et al. 2016).

Some studies have suggested that PAs may play a role in physical dormancy, for example, chemical analysis of different soybean seeds showed the content of PAs or the PAs precursor epicatechin is positively correlated with the change of hard seed percentage (Zhou et al. 2010; Vijayan et al. 2023); mass spectrometry analysis of separated seed coats revealed significantly higher contents of PAs and hydroxylated fatty acids in dormant seeds compared with nondormant seeds in pea (Hradilová et al. 2017); distribution of condensed tannins in the seed coat contributes to the seed coat impermeability in black bean (Nicolás-García et al. 2022); in low tannin seeds of faba bean, the seed coat is more prone to cracking and the seed hardness is lower with faster water absorption rate (Kantar et al. 1996). However, in the current study, the seeds of *tt8*, *f3′5'h1,* and *anr tt8* exhibited physical dormancy, and these mutants lack PAs in their seed coats, the evidence suggests that, in contrary to the above reports, the accumulation of PAs is not directly associated with physical dormancy.

### Decrease in lipid accumulation affects physical dormancy

The seed coat cuticle is made up of polyesters of fatty acid derivatives, cutin, and wax, which are commonly present in many plant tissues exposed to air (Chai et al. 2021). The lipid content in the seed coat affects its development. The lack of hydroxylated fatty acids in the seed coat of *M. truncatula* changes the permeability of the cuticle layer (Shao et al. 2007). *MtKNOX4* and *MtKCS12* play a role in the formation of seed physical dormancy by affecting the synthesis of fatty acids in the seed coat in *M. truncatula* (Chai et al. 2016, 2021). In the current study, microscopic observation of cross-sections stained with Oil red O confirmed that the *anr* mutants had fewer lipid droplets in parenchyma cells. RNA-seq analysis showed that lipid-related genes were downregulated in the mutant compared with WT at 4 stages. Metabolic analysis revealed that compared with the WT, the *tt8* mutant and the *anr tt8* double mutant, the content of glycolipids was decreased in the *anr* mutant. Glycolipids are considered important components of cell membranes, playing important roles in cell growth, differentiation, signal transduction, and chloroplast biogenesis. MGDG synthesis has an influence on cotyledon and thylakoid membranes development in *Arabidopsis*, and contributes to maintaining membrane stability and permeability under abiotic stress (Fujii et al. 2014; Zhang et al. 2016; Kong et al. 2018). Disruption of LTPG15, which codes for a glycosylphosphatidylinositol (GPI)-anchored lipid transfer protein, caused a significant decrease in the levels of fatty acids, primary alcohols, ω-hydroxy fatty acids, and α, ω-alkanediols, thereby affecting seed coat development in *Arabidopsis* (Lee and Suh 2018).

### The mechanism by which *ANR* regulates the permeability of the seed coat

Since ANR is downstream of TT8, F3′5*′*H1, and ANS in the proanthocyanidin and anthocyanin biosynthetic pathway, double mutants of *anr* and *tt8*, *f3′5′h1,* and *ans* were respectively created in *M. truncatula* through sexual hybridization. The phenotype of the *anr tt8* and *anr f3′5′h1* double mutant seeds was similar to that of *tt8* and *f3′5′h1,* respectively, namely, the seed coat was transparent and impermeable. The phenotype of the *anr ans* seeds was similar to that of *ans*, namely, the seed coat showed normal color and was impermeable. The results revealed that *tt8*, *f3′5′h1,* and *ans* were able to restore seed physical dormancy in the *anr* mutant. It is known that knockout of *TT8*, *F3′5′H1,* or *ANS* hindered the synthesis of corresponding flavonoids, thereby affecting accumulation of flavonoid-3-*O*-glucoside (Dixon and Sarnala 2020). Our study showed that compared with the WT, the *tt8* mutant and the *anr tt8* double mutant, the *anr* mutant accumulated excessive amounts of quercetin-3-*O*-glucoside, cyanidin-3-*O*-glucoside, and delphinidin-3-*O*-glucoside. The results indicate that excessive accumulation of flavonoid-3-*O*-glycosides is associated with seed physical dormancy.

The biosynthesis of different kinds of flavonoids is tissue-specific and developmentally regulated. It has been shown that flavonoids affect plant growth and development by regulating auxin transport (Dong et al. 2024). Quercetin and its derivatives are inhibitors of basipetal root auxin transport, thus affecting gravitropism and elongation growth (Lewis et al. 2011). It will be interesting to study the possible effect of auxin transport on seed coat development and hardseededness.

Flavonoids also play a role as antioxidants that inhibit lipid synthesis (Tasdemir et al. 2006; Craciunescu et al. 2023). The lack of flavonoids could be a factor causing the increase in oil content in the seed of *BnTT8* targeted *Brassica napus* mutants (Zhai et al. 2020). In the current study, a drastic increase in the most abundant flavonoids (flavonoid-3-*O*-glucosides) coupled with a significant reduction of glycolipids was a signature change in the *anr* mutant. In addition, many free and unstable flavonoids with strong oxidation properties were produced in the *anr* mutant. In the process of stabilizing these flavonoids, the synthesis of lipids may be affected, resulting in a decrease in lipid content. Thus, the causal relationship between flavonoids and lipid accumulation in the seed coat is an interesting topic that deserves further investigation.

**Figure 8.**
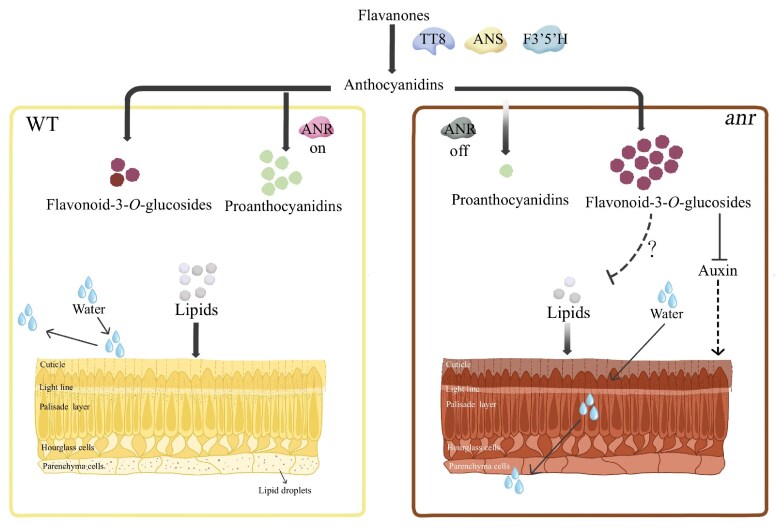
A proposed model of ANR in regulating seed physical dormancy. In the WT, normal amounts of flavonoid-3-*O*-glucosides and lipids are synthesized, leading to the formation of normal cuticles in the seed coat. In the *anr* mutant, increased accumulation of flavonoid-3-*O*-glucosides negatively affects the synthesis of lipids, leading to the formation of abnormal cuticles and thus the loss of physical dormancy. Proanthocyanidins compete with flavonoid-3-*O*-glucosides for the same substrates, while proanthocyanidins are not associated with hardseededness, the reduction of proanthocyanidins in the *anr* mutant results in an increase of flavonoid-3-*O*-glucosides. Accumulation of anthocyanins in the *anr* mutant led to a seed coat color change. ANR, anthocyanidin reductase; TT8, transparent testa 8;, F3′5′H1, flavonoid 3′,5′-hydroxylase 1; ANS, anthocyanidin synthase.

Based on the results of phenotypical, biochemical, and genetic analyses, we propose a simplified functional model of *ANR* in regulating seed physical dormancy (Fig. 8).

## Materials and methods

### Plant materials and growth conditions

*M. truncatula* ecotype R108 was used as the wild type for all experiments described in this paper. All *anr* mutant alleles were isolated from a *Tnt1* retrotransposon-tagged mutant collection of *M. truncatula*. The seeds were rubbed with sandpaper for scarification, germinated at room temperature overnight, treated at 4 °C for 24 h on filter paper, and then transferred to soil. Plants were grown under the following conditions: 26 °C/22 °C day/night temperature, 16 h/8 h day/night photoperiod, 60% to 70% relative humidity, and 150 *μ*mol·m^−2^·s^−1^ light intensity.

### *Tnt1* insertion mutant screening

Generation of the *M. truncatula Tnt1* insertional mutant population and growth of R1 seed were carried out as described previously (Tadege et al. 2008). Three *anr* mutants (NF6293, NF9161, and NF15435) were identified, one *f3′5′h1* mutant (NF3537) was identified, and the *ans* mutant (NF14857) was identified (Supplementary Fig. S15). The *tt8* mutant (NF2225) was described previously (Li et al. 2016). The *Tnt1*-specific primers and gene-specific primers were used to confirm the presence of the *Tnt1* insertion in an individual plant and its homozygous or heterozygous status (Supplementary Table S5).

### Seed germination and imbibition

For seed germination assays, 100 mature seeds were soaked in sterile water at room temperature in 3 independent experiments, and the number of germinated seeds was recorded every 6 h until 48 h. When the embryonic root was longer than the seed, it is considered that the seed has germinated. Seed imbibition was examined as previously described (Chai et al. 2021). For seed impermeability assays, 100 mature seeds were placed in sterile water, and the status of water absorption was observed and photographed using a stereo microscope with a CCD camera. For seed longevity assays, 3-year-old seeds (stored at 4 ℃, 40% to 60% RH) and freshly harvested dry seeds were compared; 100 seeds of each type were soaked in sterile water at room temperature in 3 independent experiments, and the number of germinated seeds was recorded every 6 h until 48 h.

### Generation of double mutants

The *anr-2* mutant was crossed with *tt8, f3′5′h1,* and *ans,* respectively, to generate F_1_ plants. F_1_ plants were genotyped using PCR to identify hybrids, which were then selfed to generate F_2_ plants. The double mutant phenotype was identified in a segregating population and confirmed by PCR.

### RNA extraction, RT-PCR, and RT-qPCR

Various tissues, including root, stem, leaf, flower, seed, and seed coat, were collected from greenhouse-grown plants with 3 replicates. RNA was isolated using the RNeasy Plant Mini Kit (Qiagen). cDNA reverse transcription and RT-qPCR were performed as previously described (Chai et al. 2021). Primers for quantifying the expression levels of genes are listed in Supplementary Table S5.

### Microscopic observations of the seed coat

For SEM observation, sputter coating on dry mature seeds was performed as described previously (Gou et al. 2017). To investigate the seed coat structure during development, semi-thin sections were cut as described previously (Du et al. 2021).

### Cloning and construction of binary vectors for plant transformation

*ANR*, corresponding to *Medtr4g092080* in *M. truncatula*, consisted of 6 exons and 5 introns with 2,529 base pairs. To construct the *proANR::GUS* vector, a 2.1 kb promoter region of *ANR* was amplified and cloned into pENTR/D-TOPO (Invitrogen) and confirmed by sequencing; the pENTR-*ANR*pro was transferred into the pHGWFS7 by LR recombination to create the *proANR::GUS* vector for gene expression pattern analysis. For complementation analysis, the *ANR* gene promoter and genome DNA were amplified from *M. truncatula* R108 and cloned into the pENTR/D-TOPO (Invitrogen); the pENTR-*ANR* was transferred into the pEarleyGate 301 by LR recombination to create the complementation vector. The primers used for vector construction are listed in Supplementary Table S5. Leaves of the wild type and *anr* mutant were transformed with various vectors, and transgenic plants were regenerated following the protocol described by Jiang et al. (2019).

### β-Glucuronidase staining

To determine whether there was a correlation between the *ANR* gene expression pattern and seed coat development, we examined transgenic plants that expressed *β-glucuronidase* under the control of the *ANR* gene promoter. *GUS* expression in the seed coat was analyzed at 16 DAF. GUS activity was histochemically examined, and semi-thin sections were cut as previously described (Du et al. 2021).

### Protein alignment and phylogenetic tree analysis

Predicted ANR amino acid sequences from various plant species were obtained by protein blast in GenBank (http://www.ncbi.nlm.nih.gov/) or Phytozome (https://phytozome.jgi.doe.gov/). All sequences were subsequently aligned using JALVIEW. Alignments were performed using the Protest default parameters. The phylogenetic tree was constructed by the IQ-tree program with maximum likelihood.

### RNA-seq analysis

Seed coat tissues were sampled with 3 biological replicates at 12, 16, 20, and 24 DAF. At each stage, seed coats were gently hand-dissected from seeds on ice, immediately frozen in liquid nitrogen, and stored at −80 °C for RNA-seq analysis and metabolite profiling.

RNA quality was determined using a 2100 Bioanalyzer (Agilent) and quantified using ND-2000 (NanoDrop Technologies). RNA-seq libraries were sequenced using an Illumina HiSeq xten/NovaSeq 6000 sequencer (Illumina, San Diego, CA, USA). The clean reads were mapped and annotated following the reference genome (https://medicago.toulouse.inrae.fr/MtrunR108_HiC/) with orientation mode using HISAT2 software (http://ccb.jhu.edu/software/hisat2/index.shtml). Differential expression analysis was performed using DESeq2 with a *Q*-value ≤ 0.05, and DEGs with |log2FC| > 1 and *Q*-value ≤ 0.05 was considered significant DEGs. KEGG functional enrichment and pathway analyses were carried out using Goatools (https://github.com/tanghaibao/Goatools) and KEGG (KEGG – Table of Contents).

### Metabolite profiling

Seed coat sample extracts were analyzed for lipid and flavonoid metabolites using the UPLC-ESI-MS/MS system (UPLC, ExionLC AD, https://sciex.com.cn/; MS, Applied Biosystems 6500 Triple Quadrupole, https://sciex.com.cn/). Each sample size is 0.5 g. Details of analytical method, including chemicals used, sample preparation, UPLC, and ESI-MS/MS conditions, etc., were described in the Supplementary Methods and Table S6.

### Statistical analysis

Two-tailed Student's *t*-test was used to analyze statistical data (*P < 0.05, **P < 0.01). Statistical analysis was conducted using the SPSS software.

### Accession numbers

Sequence data from this article can be found in the GenBank/EMBL data libraries under accession numbers: MtANR (Medtr4g092080), MtTT8 (Medtr1g072320), MtF3′5′H1(Medtr3g436390), and MtANS (Medtr5g011250).

## Supplementary Material

kiaf525_Supplementary_Data

## Data Availability

The data underlying this article are available in the article and in its online supplementary material.

## Supplementary data

The following materials are available in the online version of this article.

**Supplementary Movie S1.** Imbibition of wild-type (WT) and *anr* mutant (*anr*-2) seeds in water.

**Supplementary Movie S2.** Imbibition of wild-type, *anr*-1, and *anr*-3 seeds in water.

**Supplementary Figure S1.** Expression profile of *ANR* in different plant organs *in Medicago truncatula*.

**Supplementary Figure S2.** Amino acid alignment of ANRs in leguminous species.

**Supplementary Figure S3.** Phylogenetic analysis of 19 ANR proteins in different legume species.

**Supplementary Figure S4.** Test of seed impermeability.

**Supplementary Figure S5.** SEM analysis of different parts of the mature seed coat of WT and *anr*.

**Supplementary Figure S6.** Cross-sections of seed coats of 20 DAF seeds and mature seeds in WT and *anr*.

**Supplementary Figure S7.** Content of MGDG, DGDG, and SQDG of different chain lengths.

**Supplementary Figure S8.** Content of different compounds in the flavonoid pathway in *anr* and WT seed coats of *M. truncatula*.

**Supplementary Figure S9.** Venn diagram of anthocyanins.

**Supplementary Figure S10.** Generation of the *anr tt8* and *anr f3′5′h1* double mutants and the subsequent progeny segregation analysis.

**Supplementary Figure S11.** PAs content in WT, *anr*, *tt8,* and *anr tt8* seed coats of *M. truncatula*.

**Supplementary Figure S12.** Transcriptome analysis between WT and *anr* in *M. truncatula*.

**Supplementary Figure S13.** GO enrichment analysis of the differentially expressed genes at 12, 16, 20, and 24 DAF.

**Supplementary Figure S14.** Germination rate of WT, *anr,* and complementation line seeds.

**Supplementary Figure S15.** Schematic representation of *TT8*, *ANS,* and *F3′5′H1* gene structure and Tnt1 insertion sites.

**Supplementary Table S1.** Content of different classes of lipids.

**Supplementary Table S2.** Content of the top 30 lipid monomers in WT and *anr*.

**Supplementary Table S3.** Transcriptome data before and after filtering.

**Supplementary Table S4.** Details of candidate genes.

**Supplementary Table S5.** Primers used in this study.

**Supplementary Table S6.** Details of the conditions used to identify metabolites.

## Dive Curated Terms

The following phenotypic, genotypic, and functional terms are of significance to the work described in this paper:

KCS12 Gramene: AT2G28630
KCS12 Araport: AT2G28630

LDOX Gramene: AT4G22880
LDOX Araport: AT4G22880

TT8 Gramene: AT4G09820
TT8 Araport: AT4G09820

seed AmiGo: PO:0009010

flavonoids CHEBI: CHEBI:72544

NCBI Gramene: https://www.ncbi.nlm.nih.gov
NCBI Araport: https://www.ncbi.nlm.nih.gov

flavonoid CHEBI: CHEBI:47916
