# Supplementary material for: ANTHOCYANIDIN REDUCTASE promotes physical dormancy in Medicago truncatula seeds
Source: Plant Physiol. 2025 Oct 17;199(3):kiaf525. doi: 10.1093/plphys/kiaf525 (PMC12596370; doi:10.1093/plphys/kiaf525)
Supplement: kiaf525_Supplementary_Data [file kiaf525_supplementary_data.zip › Supplementary Data (3).pdf]

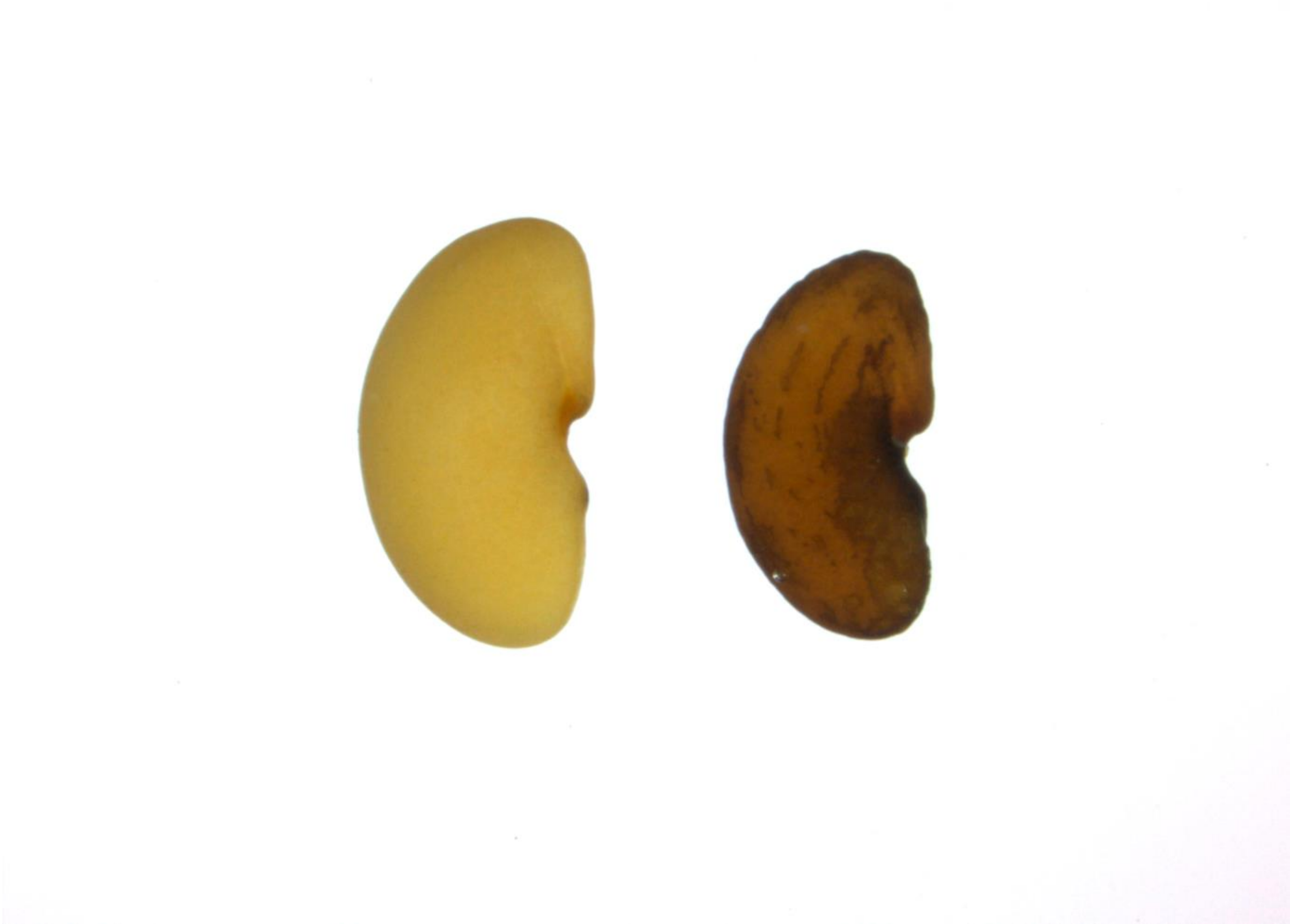

Supplementary Movie S1. Imbibition of wild-type (WT) and *anr* mutant (*anr*-2) seeds in water. Images were captured at 15 s intervals and played back at 25 fps.

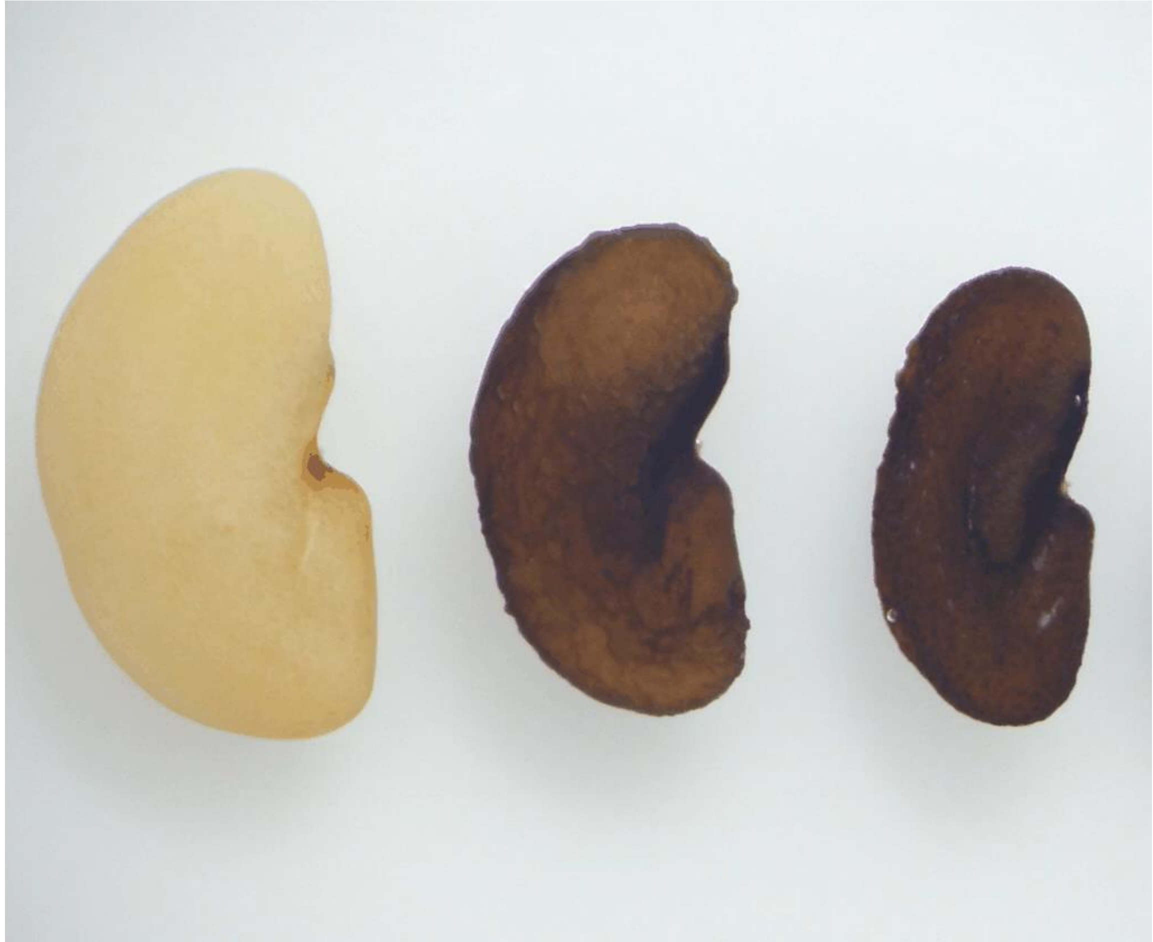

Supplementary Movie S2. Imbibition of the wild-type, *anr1* and *anr-3* seeds in water. Images were captured at 30 s intervals and played back at 50 fps.

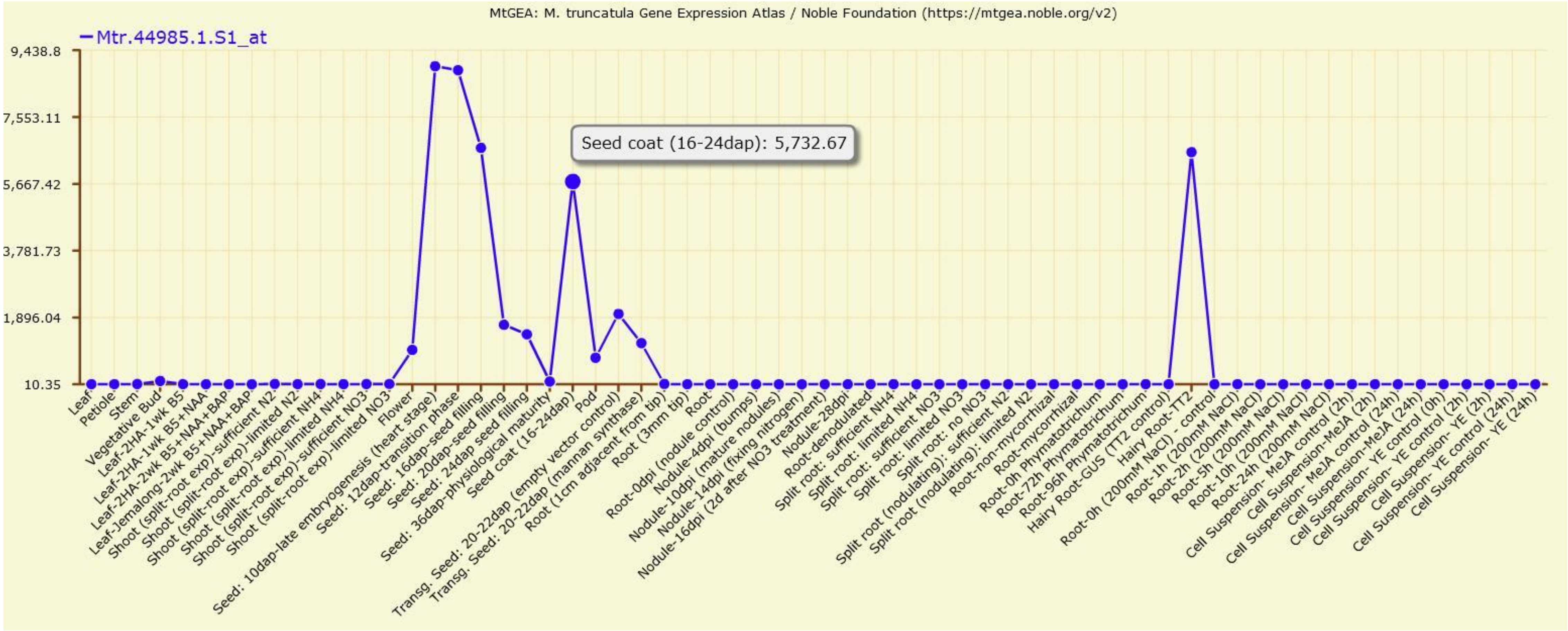

Supplementary Figure S1 Expression profiles of *ANR* in different plant organs *in Medicago truncatula*. The y-axis represents the expression level, with three replicates for each sample (mtgea.noble.org/v2).

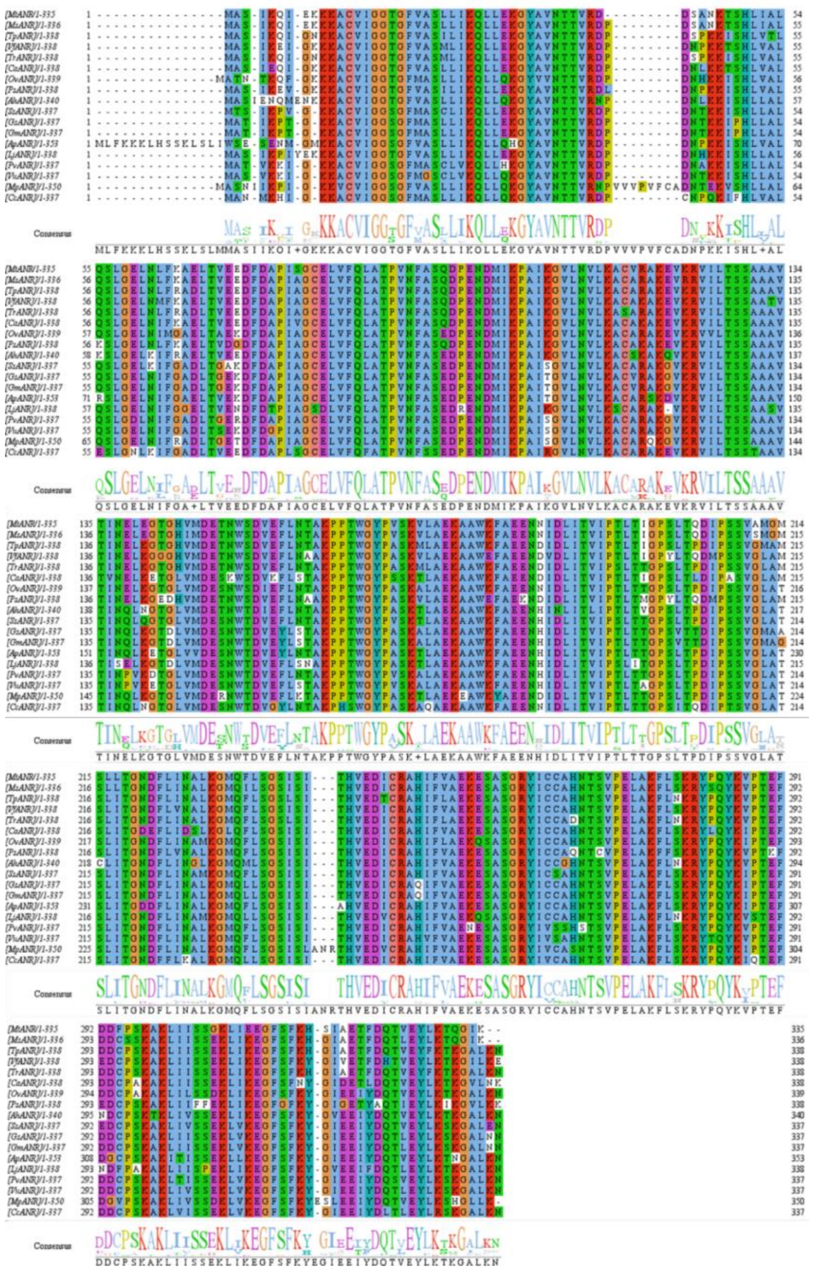

Supplementary Figure S2 Amino acids alignment of ANRs in leguminous species.

Red represents phenylalanine (F), tryptophan (W), and tyrosine (Y); blue represents lysine (K), arginine (R), and histidine (H); purple represents aspartic acid (D) and glutamic acid (E); green represents serine (S), threonine (T), asparagine (N), and glutamine (Q); orange represents alanine (A), valine (V), leucine (L), isoleucine (I), methionine (M), and proline (P); and gray represents glycine (G) and cysteine (C).

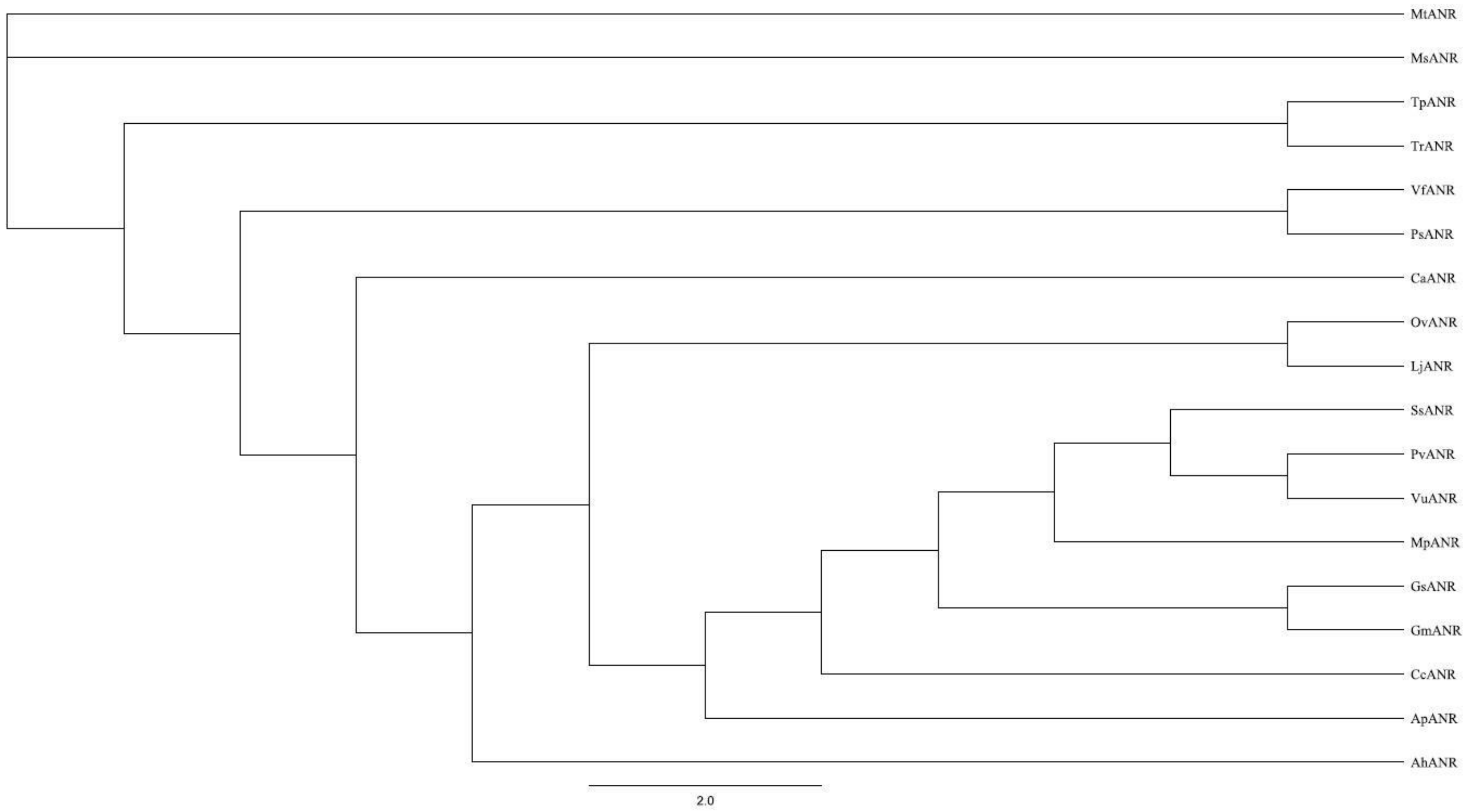

Supplementary Figure S3 Phylogenetic analysis of 19 ANR proteins in different legume species.

[MsANR, ADK95116.1] *Medicago sativa*; [TpANR, PNY07320.1] *Trifolium pratense*; [VfANR, AJK93561.1] *Vicia faba*; [TrANR, ADD09575.1] *Trifolium repens*; [CaANR, XP_004504947.1] *Cicer arietinum*; [OvANR, AJF94633.1] *Onobrychis viciifolia*; [PsANR, AII26022.1] *Pisum sativum*; [AhANR, XP_025689942.1] *Arachis hypogaea*; [SsANR, TKY59382.1] *Spatholobus suberectus*; [GsANR, KAG4999436.1] *Glycine soja*; [GmANR, NP_001241913.3] *Glycine max*; [ApANR, XP_027348380.1] *Abrus precatorius*; [LcANR, ABM90632.1] *Lotus corniculatus*; [PvANR, ARB08607.1] *Phaseolus vulgaris*; [VuANR, XP_027917926.1] *Vigna unguiculata*; [MpANR, RDX81948.1] *Mucuna pruriens*; [CcANR, XP_020238816.1] *Cajanus cajan.* The scale unit is substitutions per site.

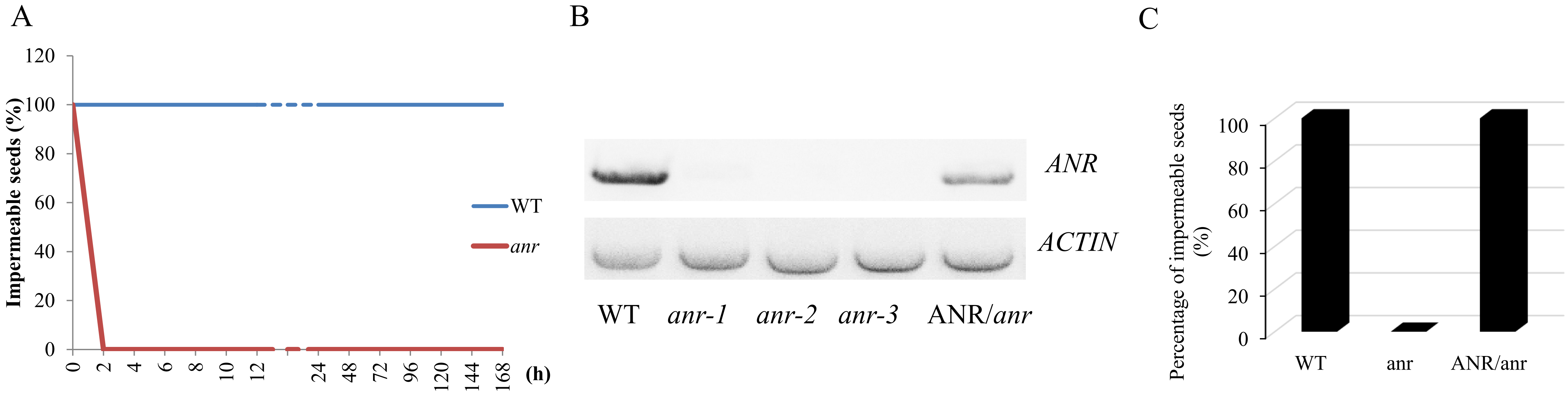

Supplemental Figure S4 Test of seed impermeability. A: Percentage of impermeable (hard) seeds in water at multiple points. h, hour; B: RT-PCR analysis of *MtANR* transcript levels in WT, *anr* mutants and the complementation line, *ACTIN* used as the control. C:Seed permeability analysis of WT, *anr* mutant and the complementation line.

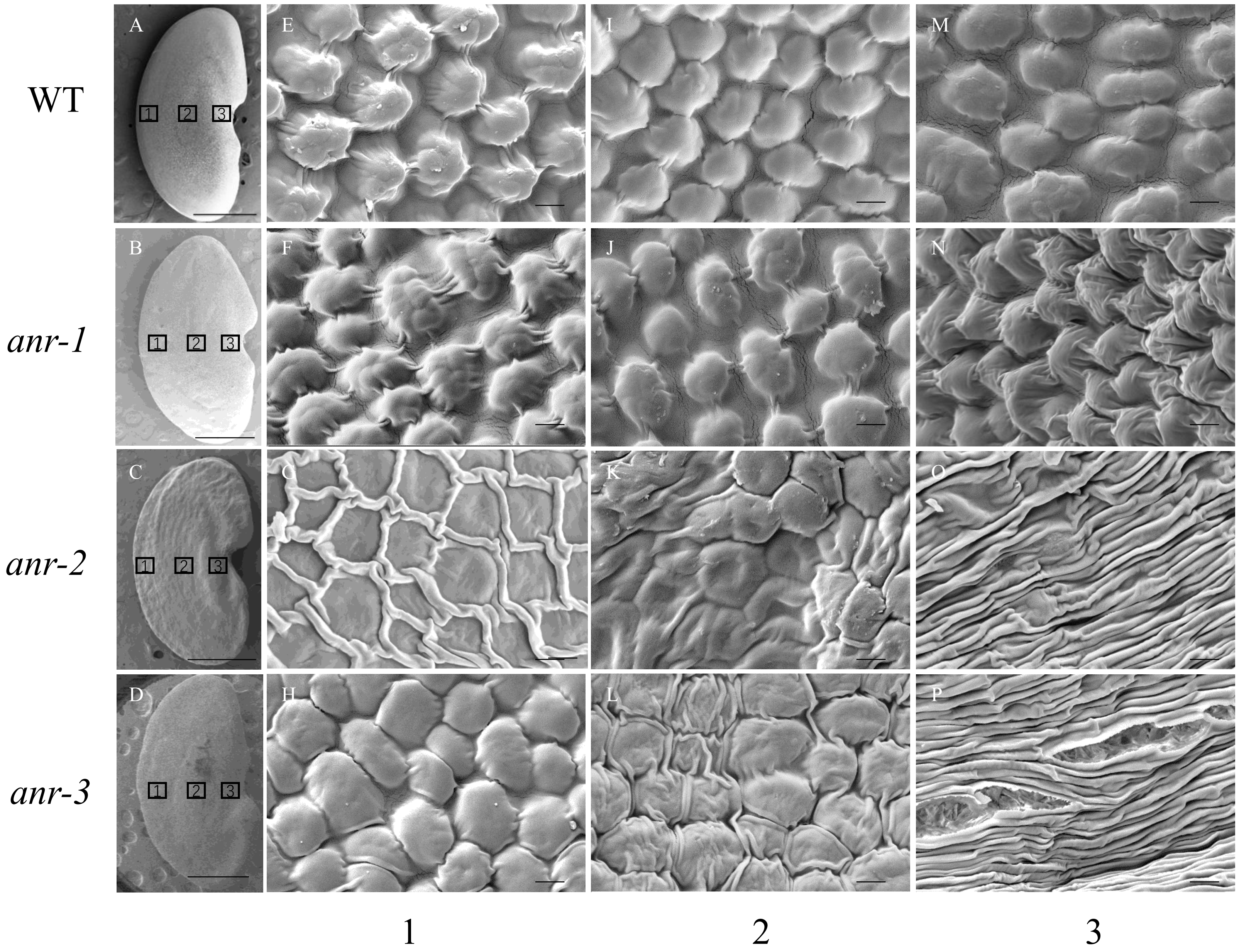

Supplementary Figure S5 Scanning electron microscopy (SEM) analysis of different parts of the mature seed coat of WT and *anr*.
1, the edge away from the seed hilum. 2, the middle of the seed. 3, near the hilum. Scale bar:. A-D: Scale bar = 1 mm, E-P: Scale bar = 2 μm.

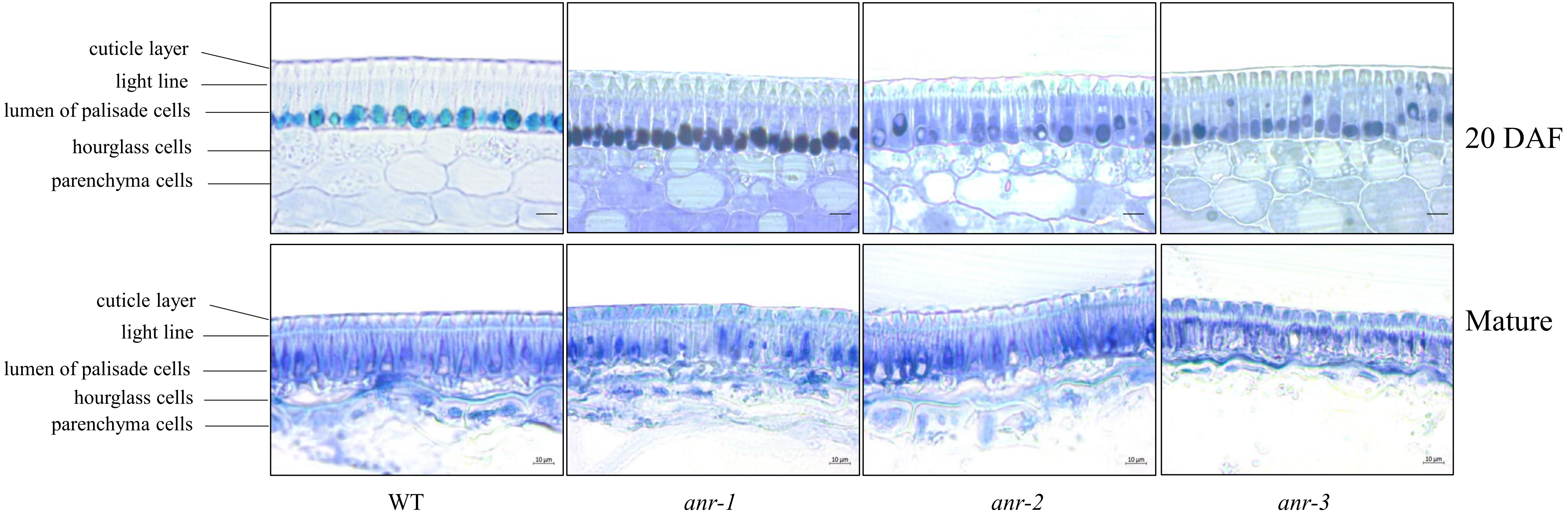

Supplementary Figure S6 Cross-sections of seed coats of 20 DAF seeds and mature seeds in WT and *anr*. Scale bar = 10 μm

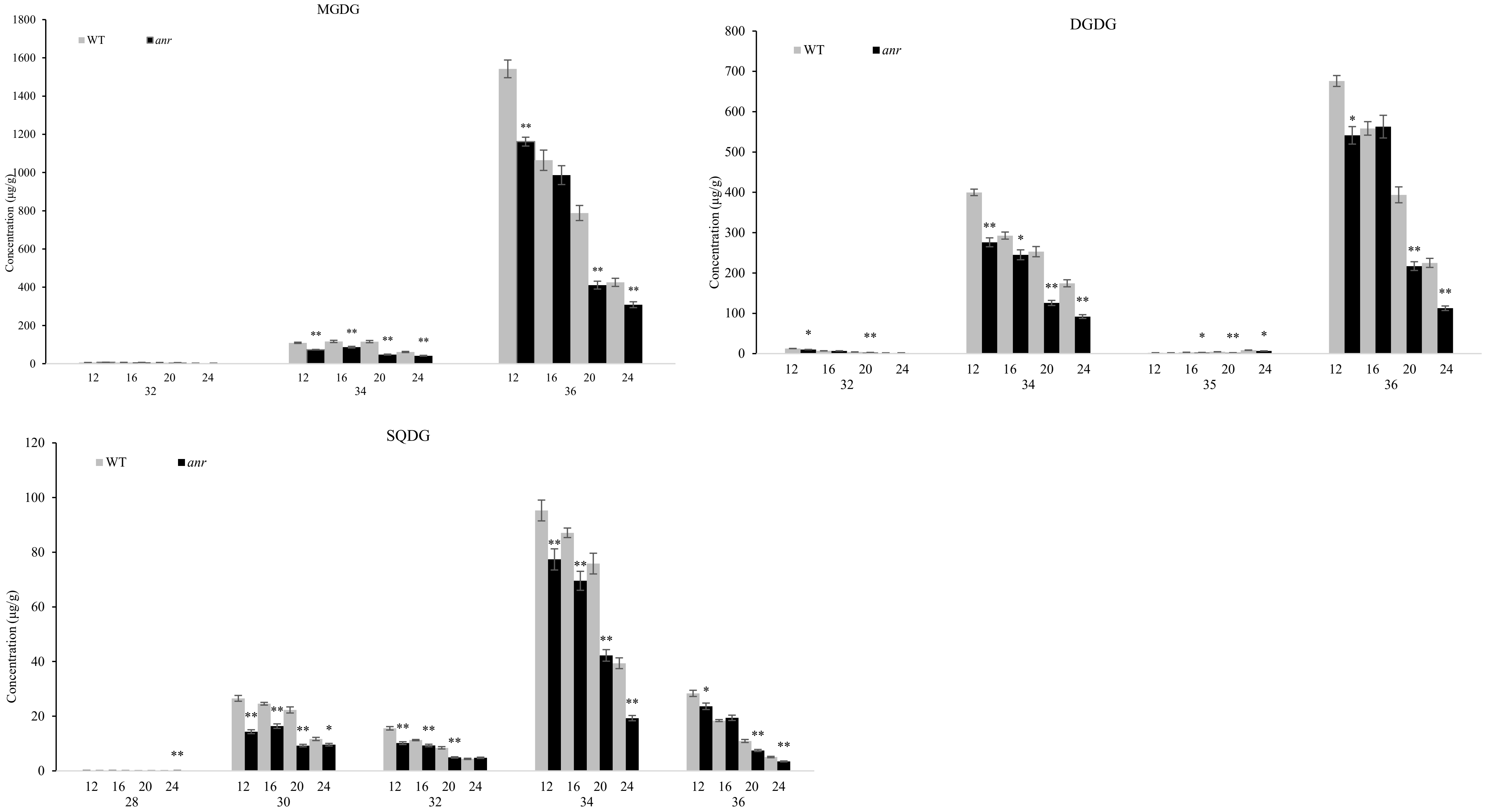

Supplementary Figure S7 Content of MGDG, DGDG and SQDG of different chain lengths.

Error bars represent SD, asterisks indicate significant differences between WT and *anr* mutant using two-tailed unpaired Student's t-test in three independent experiments (**P*<0.05; ***P*<0.01).

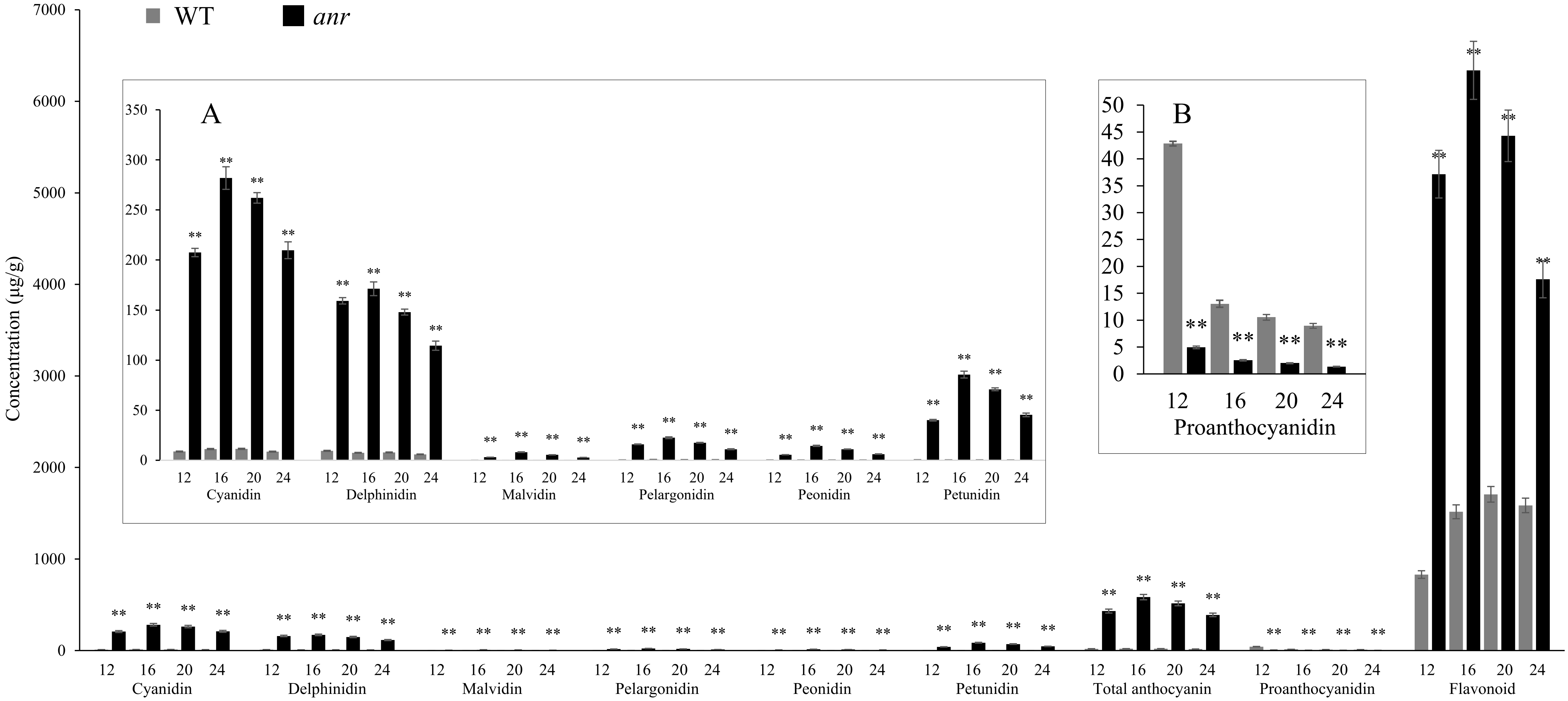

Supplementary Figure S8 Content of different compounds in the flavonoid pathway in *anr* and WT seed coats of *M. truncatula*.

A: Content of different type of anthocyanins. B: Content of PAs. Error bars represent SD, asterisks indicate significant

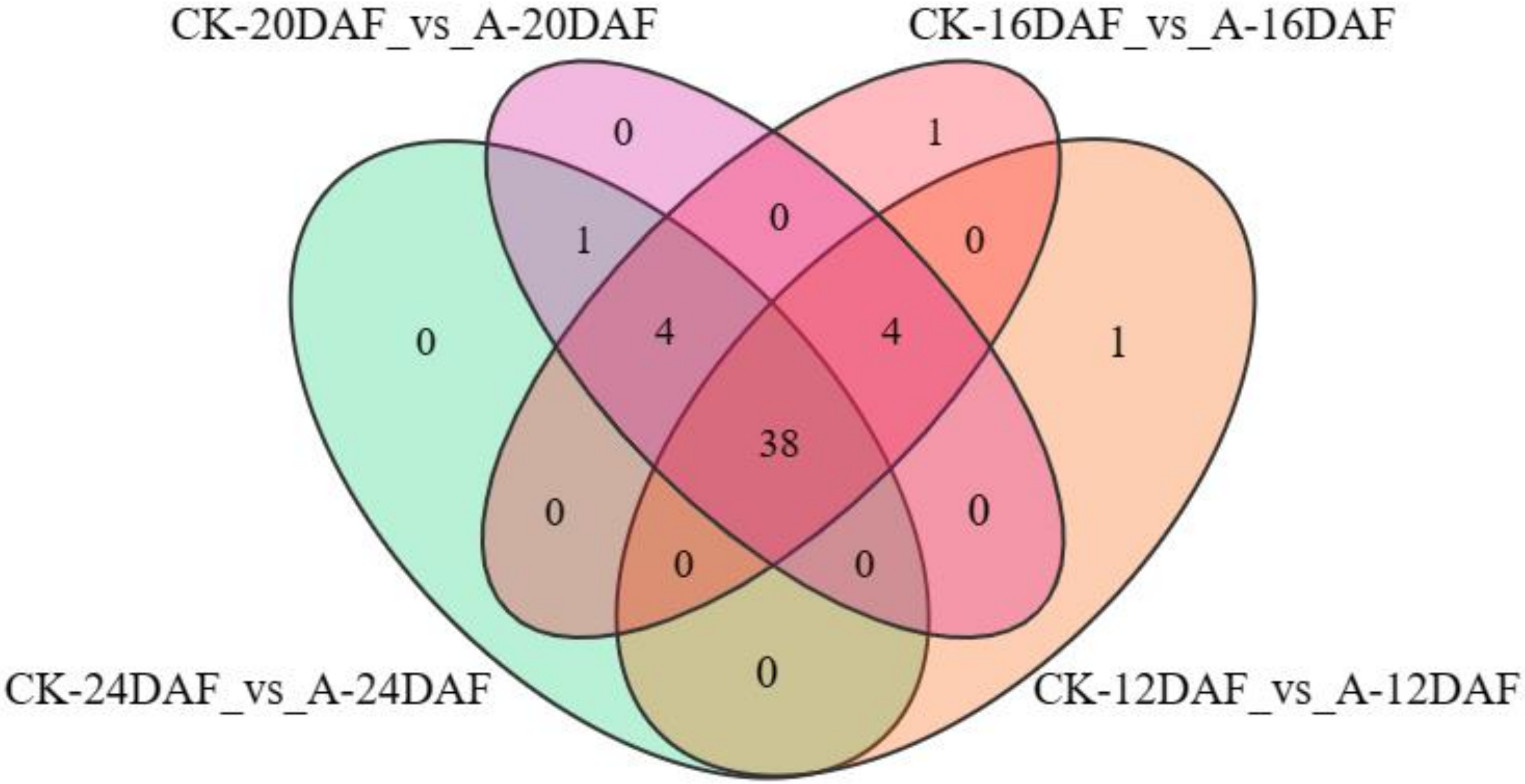

Supplementary Figure S9 Venn diagram of anthocyanins.
CK represents wild type, A represents anr mutant.

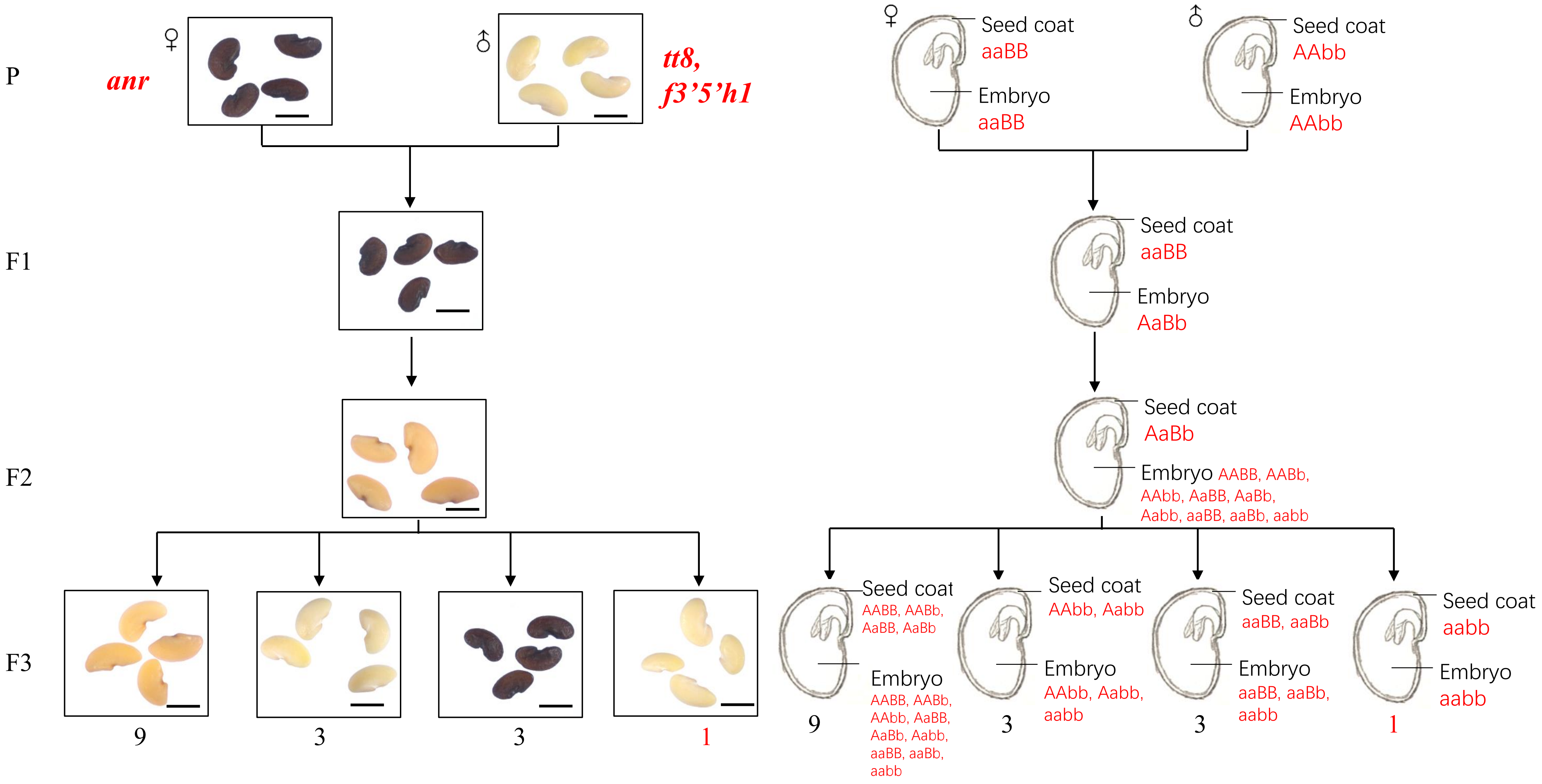

Supplementary Figure S10 Generation of the *anr tt8* and *anr f3'5'h1* double mutants and the subsequent progeny segregation analysis. A/a stand for *ANR*, B/b stand for *TT8* or *F3'5'H1*, upper case represents normal, lower case represents mutation, scale bar = 1 mm.

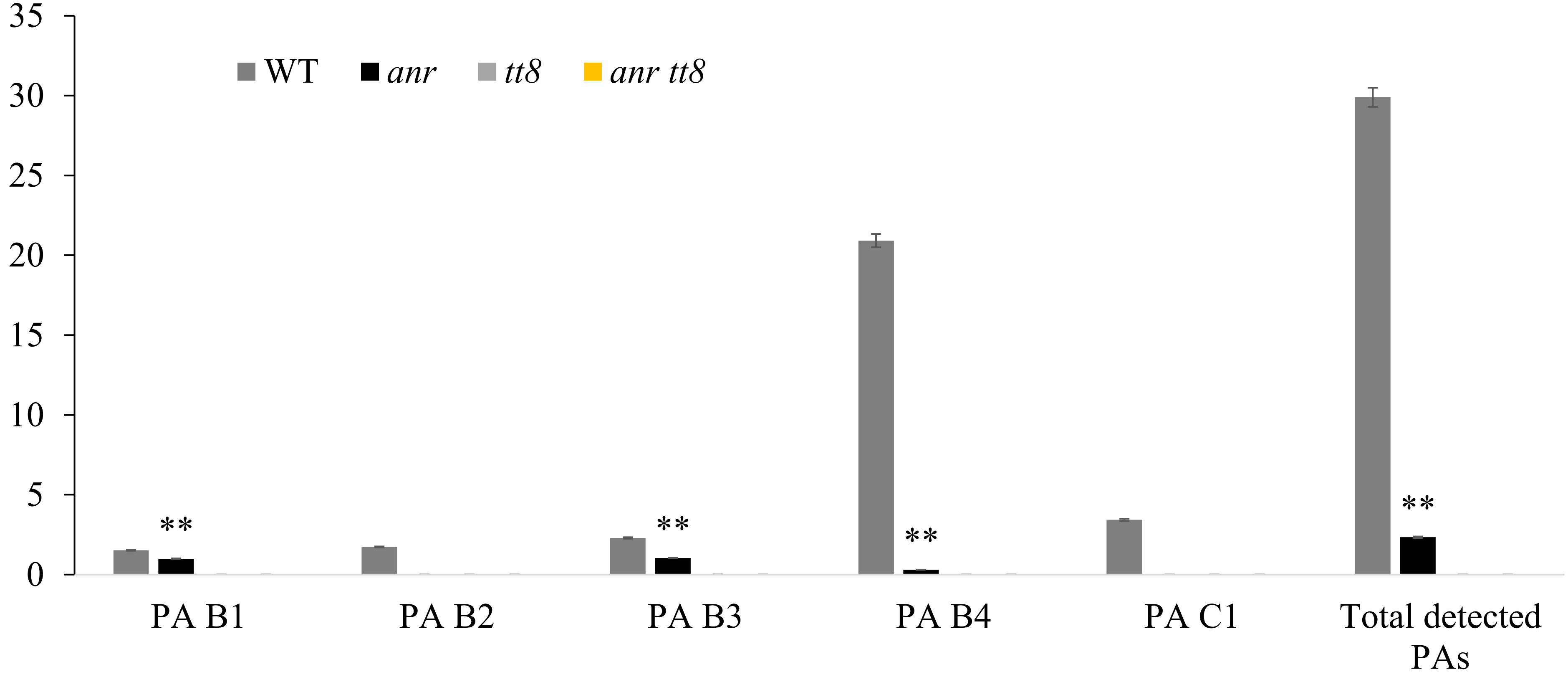

Supplementary Figure S11 PAs contents in WT, *anr*, *tt8* and *anr tt8* seed coats of *M. truncatula*. Inset: error bars represent SD, asterisks indicate significant differences between WT and *anr* mutant using two-tailed unpaired Student's t-test (**P*<0.05; ***P*<0.01). PAs: proanthocyanidins.

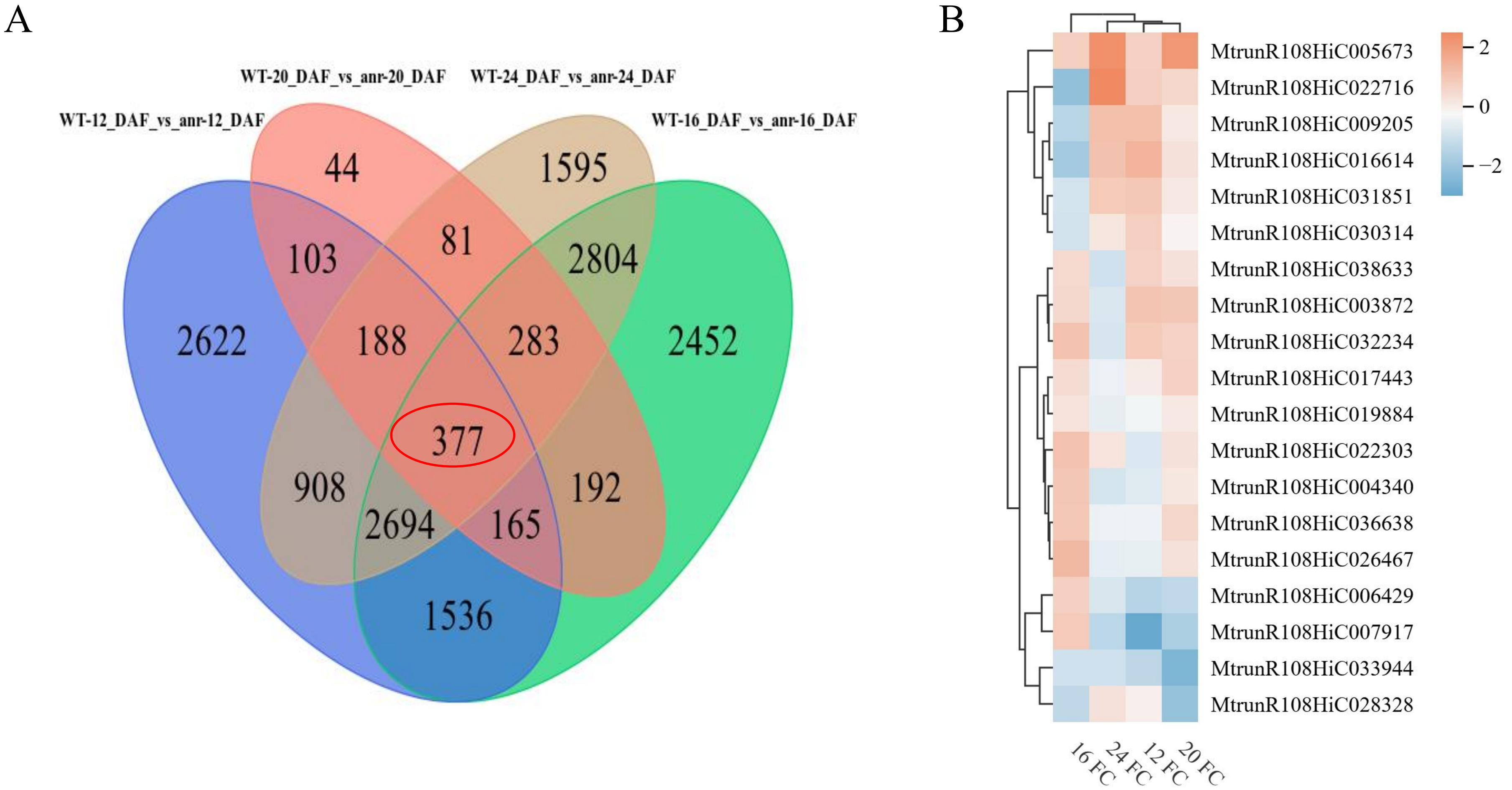

Supplementary Figure S12 Transcriptome analysis between WT and *anr* in *M. truncatula*.
A: Venn-diagram showing numbers of genes that are differentially expressed in seed coat at 12, 16, 20 and 24 DAF stages, compared to WT. B: Cluster heatmap of candidate genes, Orange represents increase, blue represents decrease.

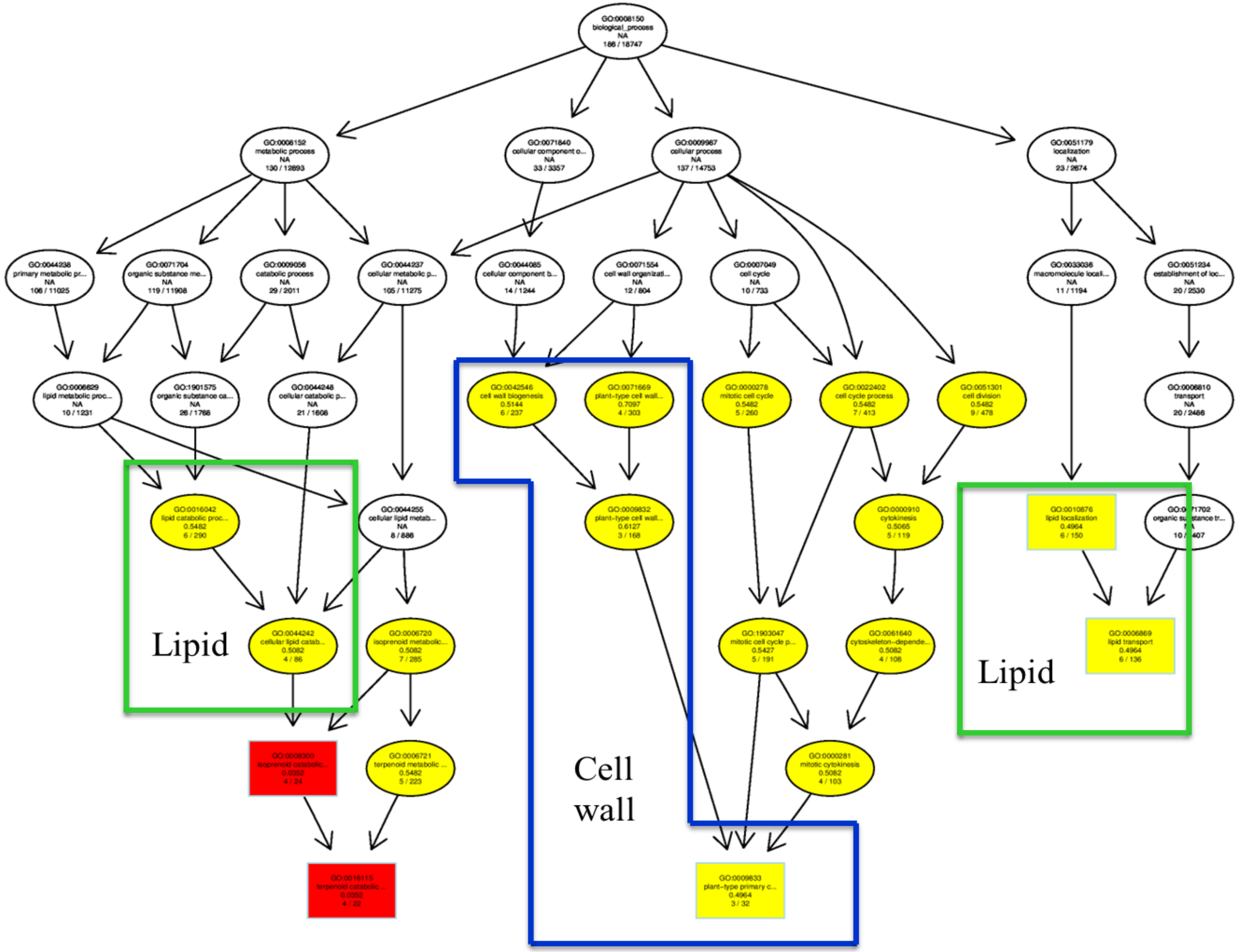

Supplementary Figure S13 GO enrichment analysis of the differentially expressed genes at 12, 16, 20 and 24 DAF. Green represents pathways related to lipid; blue represents pathways related to cell wall.

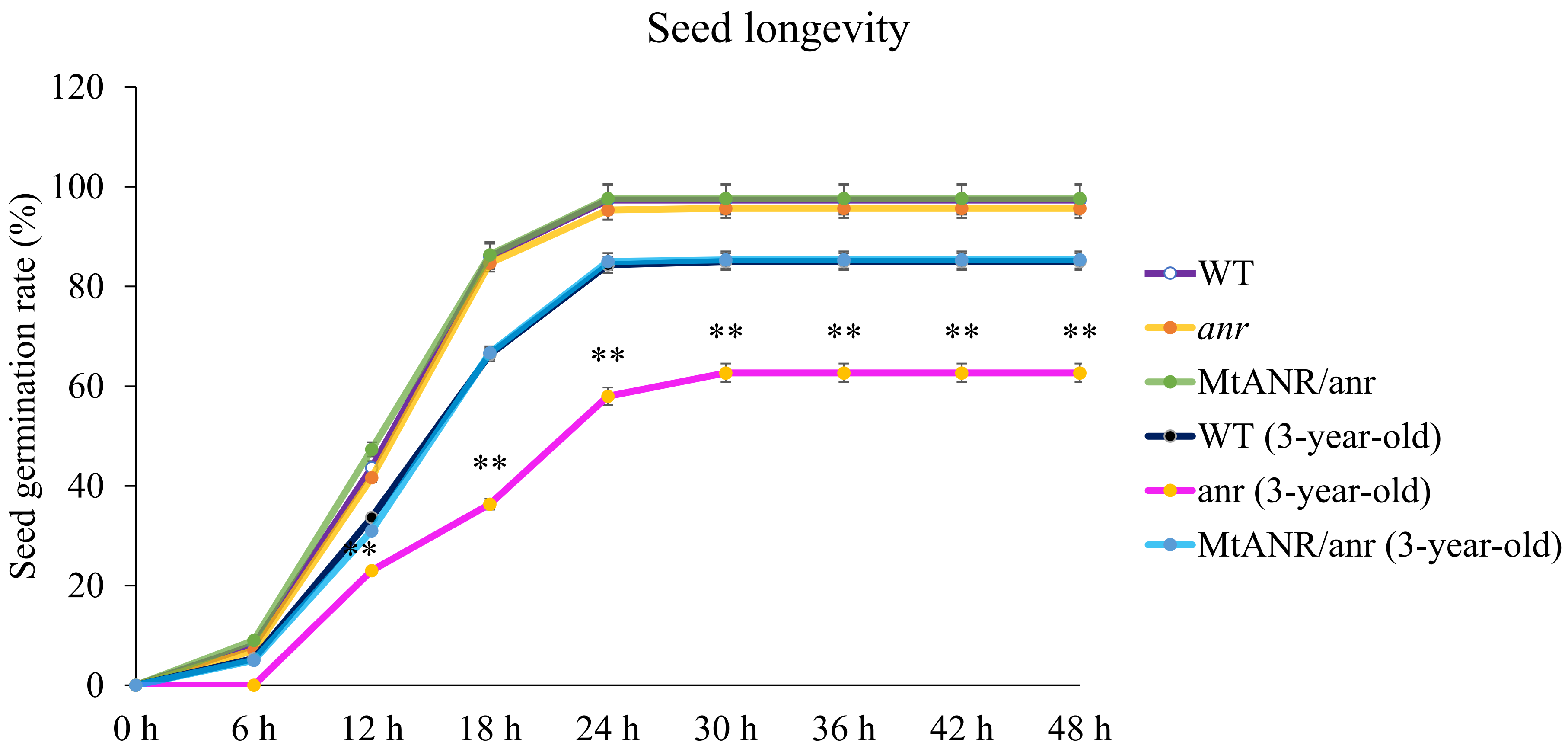

Supplementary Figure S14 Germination rate of WT, *anr* and complementation line seeds. Statistical analyses were performed relative to the respective wild type. Error bars represent SD, asterisks indicate significant differences using two-tailed unpaired Student's t-test (**P*<0.05; ***P*<0.01).

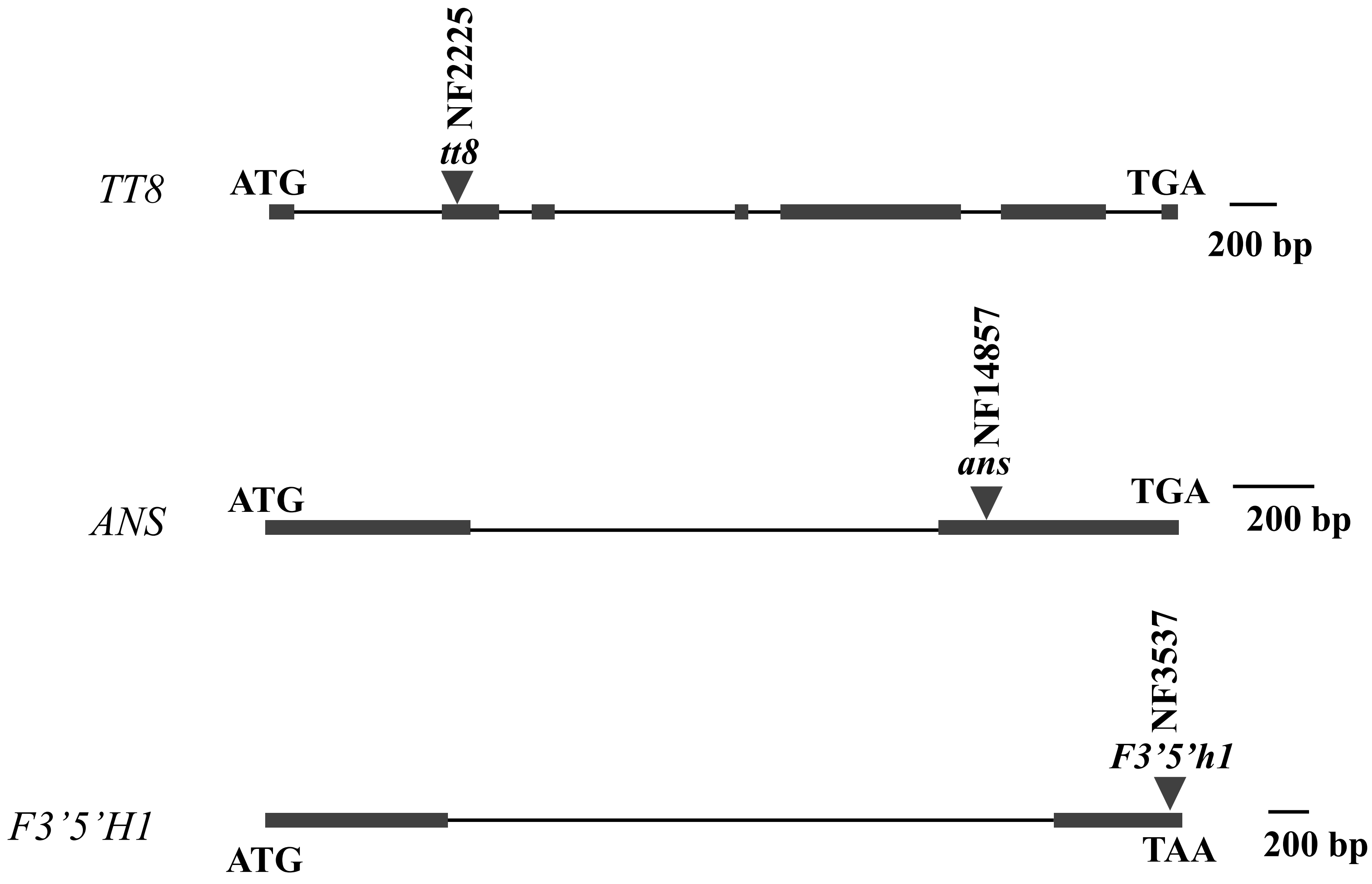

Supplementary Figure S15 Schematic representation of *TT8*, *ANS* and *F3'5'H1* gene structure and Tnt1 insertion sites. filled black boxes represent exons and lines between them denote introns.
