# Supplementary material for: ANTHOCYANIDIN REDUCTASE promotes physical dormancy in Medicago truncatula seeds
Source: Plant Physiol. 2025 Oct 17;199(3):kiaf525. doi: 10.1093/plphys/kiaf525 (PMC12596370; doi:10.1093/plphys/kiaf525)
Supplement: kiaf525_Supplementary_Data [file kiaf525_supplementary_data.zip › Supplementary video legends.docx]

Supplementary Movie S1. Imbibition of wild-type (WT) and *anr* mutant (*anr*-2) seeds in water.

Images were captured at 15s intervals and played back at 25 fps.

Supplementary Movie S2. Imbibition of the wild-type, *anr1* and *anr-3* seeds in water.

Images were captured at 30s intervals and played back at 50 fps.
